# Fos-induced osteosarcoma growth causes a cachexia-like phenotype in mice and correlates with high Fgf21 serum levels

**DOI:** 10.1186/s40170-025-00417-y

**Published:** 2026-02-03

**Authors:** Julia Luther, Armelle Carreau, Christina Baldauf, Mona Neven, Till Koehne, Michael Amling, Thorsten Schinke

**Affiliations:** 1 Department of Osteology and Biomechanics (IOBM), University Medical Center Hamburg-Eppendorf, Martinistraße 52, 20251 Hamburg, Germany; 2 Department of Orthodontics, University of Leipzig Medical Center, Liebigstraße 12, Haus 1, 04103 Leipzig, Germany

**Keywords:** Osteosarcoma, Fos, AP-1, Cachexia, Fgf21, Metabolism

## Abstract

**Background:**

The dimeric AP-1 transcription factor has been described as a regulator of bone metabolism, but also of adipocyte biology. More specifically, its family members adopt specific functions in the regulation of adipocyte differentiation and adipose tissue hypoxia, but also in controlling lipolysis, energy expenditure and insulin sensitivity.

**Methods:**

In this study, we analyzed the role of the AP-1 family member Fos, whose ubiquitous over-expression in transgenic mice has been described to cause osteosarcoma formation, on adipose tissue, glucose and lipid metabolism. More specifically, we analyzed the metabolic phenotype of the respective mouse model by histological analyses, monitoring gene expression, determination of serum parameters and ex vivo adipogenesis assays.

**Results:**

We show that *Fos*Tg mice additionally display an age-dependent loss of white adipose tissue, which is associated with reduced adipocyte size. Quantitative real-time PCR analyses of white adipose tissue as well as in vitro studies using adipocyte-derived stromal cells excluded a cell-autonomous defect in adipocyte differentiation. However, *Fos*Tg mice displayed low circulating glucose levels along with increased glucose tolerance and insulin sensitivity. By proteomic analysis we identified elevated serum levels of fibroblast growth factor 21 (Fgf21) in *Fos*Tg mice which could explain the observed reduced hepatic lipogenesis and white adipose tissue mass. Most importantly, we demonstrated that this metabolic phenotype is dependent on osteosarcoma formation by crossing the *Fos*Tg mice with mice deficient for *Rsk2*, a kinase necessary for tumor growth. Moreover, we could show a direct correlation between tumor burden and Fgf21 serum levels in our mouse model.

**Conclusion:**

In summary, our data identify Fgf21 as a potential regulator of glucose and lipid metabolism in *Fos* transgenic osteosarcoma-bearing mice, thereby supporting its essential function in the regulation of the metabolic phenotype associated with cancer cachexia.

**Supplementary Information:**

The online version contains supplementary material available at 10.1186/s40170-025-00417-y.

## Introduction

The transcription factor AP-1 is formed by heterodimerization of one of the four Fos family members (Fos, FosB, Fra1 and Fra2) with one of the three Jun family members (Jun, JunB and JunD) or by homodimerization of members of the Jun family. The expression and activity of the family members are regulated by a variety of extracellular stimuli including growth factors, pro- and anti-apoptotic agents or differentiation signals. Consequently, AP-1 has been implicated in the regulation of cell proliferation and apoptosis and it is involved in the differentiation of various cell types as well as in tumor development [1]. Genetic analysis using gain- or loss-of-function experiments in mice, further revealed the key function played by each AP-1 member in skeletal development or in the regulation of bone remodeling necessary for post-developmental bone maintenance [2]. Specifically, gain-of-function of any of the Fos members has generated phenotypes affecting bone-forming cells, i.e. osteoblasts. For instance, the main consequence of an ubiquitously overexpressed short isoform of FosB (ΔFosB), of Fra1 (encoded by Fosl1) or of Fra2 (encoded by Fosl2) is increased osteoblast differentiation leading to osteosclerosis [3–5]. The function of Fra1 and Fra2 as physiological regulators of bone formation has been confirmed by the analysis of mouse deficiency models, which developed osteopenia due to decreased osteoblast function [5, 6].

In contrast to other AP-1 members, ubiquitous over-expression of Fos (originally called c-Fos) in transgenic mice leads to osteosarcoma formation due to the transformation of chondro-osteoblastic cells, a phenocopy of the tumorigenic activity of the viral oncogene v-fos [7, 8]. The relevance of Fos in tumor formation is confirmed by its increased expression in human osteosarcomas [9, 10]. In H2kb-FosTg mice, Fos transgene expression occurs postnatally in a tissue-specific manner. However, tumor formation is restricted to bone tissue where transgene expression starts at 2–3 weeks of age, leading to osteosarcoma formation in all bones with 100% penetrance [7].

Cancer cachexia, affecting approximately 80% of cancer patients, is a catabolic syndrome, characterized by a progressive loss of muscle mass, fat mass and body weight, that cannot be completely reversed by nutritional supplementation [11]. During tumor-associated anorexia-cachexia, Fos induction was detected in the forebrain [12]. Furthermore, elevated IL-6 levels associated with increased Fos expression regulating cancer cachexia were detected in the area postrema [13]. In addition, the AP-1 signaling cascade has been implicated in muscle wasting [14, 15], in particular as persistent Fos expression in muscle progenitor cells disrupts myogenic differentiation [16], while blocking AP-1 restores muscle mass in tumor-bearing rats [15, 17]. Finally, Fos has been described to be induced when stimulated by growth differentiation factor 15 (GDF15), a factor implicated in cachexia [18].

Fgf21 is a recently discovered factor that is proposed as a prognostic biomarker associated with a poor prognosis in various cancers [19–21]. Its expression has been described to be increased in several types of cancer, but the effect seems tumor-, context- and dose-dependent. For instance, Fgf21 has been described to inhibit pancreatic cancer cell proliferation [22] and pancreatic inflammation [23] and furthermore delayed liver tumor development [24]. In other models, Fgf21 secretion by tumors promoted the growth of lung cancer cell lines [25] as well as tumor progression [20, 26]. Originally however, Fgf21 has been described as an endocrine factor primarily produced in the liver, where its expression is increased in response to starvation. Nonetheless, Fgf21 is also expressed in pancreas, white adipose tissue and muscle and it plays a crucial role in regulating energy expenditure, glucose and lipid metabolism. Gain- and loss-of-function studies, as well as pharmacological administration of Fgf21 in mice have additionally demonstrated its function as a regulator of insulin sensitivity and adipose tissue mass [27–29].

In the present study, we investigated the effects of ubiquitous Fos over-expression in mice, which causes osteosarcoma formation, on adipose tissue, glucose and lipid metabolism. Our analyses demonstrate a decreased adipose tissue mass and an increased glucose tolerance and insulin sensitivity in *Fos*Tg mice. Proteomic analysis identified Fgf21 as a central mediator that can cause the metabolic alterations associated with the cachexia-like phenotype. Importantly, we could show a correlation between Fgf21 levels and tumor burden, indicating that Fgf21 has an essential function in the regulation of metabolic effects associated with cancer cachexia.

## Materials and methods

### Mice

The *Fos*Tg mice (H2-*c-*fos-LTR), described by Ruther et al. [30], were crossed in a C57BL/6 background. RSK2 knockout mice were previously described [31, 32]. Genotyping was performed using the following primers: H2cfos up 5’-AGT CTG GCC TGC GGG TCT CT-3’, H2cfos down 5’-GTC GGC TGG GGA ATG GTA GTA GG-3’, Rsk2 up 5´-TTG TTG GTT TAC TTT CTT TCG GTC TG-3´ and Rsk2 down 5’-AAG ATG ATT GCT TTG CTT AGT TTA-3’. Mice were kept under standard diet (1328P, Altromin Spezialfutter GmbH&Co. KG) ad libitum in a specific pathogen-free environment with a 12 h light/dark cycle and 20–24 °C ambient temperature. A maximum of 6 mice was housed in individually ventilated cages. All animal experiments were approved by the local animal facility and by the ‘Amt für Gesundheit und Verbraucherschutz’ (Org_633, Org_984, N22/059).

### Tolerance tests

To determine the glucose tolerance, overnight fasted mice were intraperitoneally injected with 2 mg/g body weight of glucose. For insulin tolerance tests, mice fasted for 6 h were injected with 0.75U insulin. Blood glucose levels were measured by incision of the tail vein using a glucometer (Ascentia Elite, Bayer). These animal experiments were approved by the local animal facility and by the ‘Amt für Gesundheit und Verbraucherschutz’ (G16/073).

### RNA analyses

RNA was isolated using Trifast Reagent (Peqlab) according to the manufacturer’s instructions and RNA was reversed transcribed using the Verso cDNA kit (Thermo Scientific). Q-PCR analyses were performed using predesigned TaqMan gene expression assays (Thermo Scientific) or SybrGreen (Applied Biosystems) with self-designed primers in a StepOnePlus system (Thermo Scientific). Results were normalized to the level of *GapDH* (adipose tissue, bone tissue, tumor and cell culture experiments) or *Hprt* (liver).

### Protein analyses

Serum protein concentrations were quantified by Elisas: leptin and insulin (CrystalChem), adiponectin (Invitrogen), Fgf21 (R&D) and osteocalcin (AlfaAesar). Triglycerides and non-esterified fatty acids were quantified by colorimetric assays (Sigma and Wako Diagostics, respectively).

Total protein and nuclear protein extracts were isolated as previously described [33, 34]. In brief, total protein was extracted by incubation of cells with RIPA buffer (150 mM NaCl, 2 mM EDTA, 10 mM sodium phosphate, 1% NP-40, 1% sodium desoxycholat, 0.1% SDS) for 15 min, followed by 10 min of centrifugation at 16.000 rpm and 4 °C. For nuclear extracts, protein was isolated by addition of 450 µl buffer A (50 ml consist of: 500 µl HEPES (1 M), 250 µl KCl (2 M), 10 µl EDTA (0.5 M), 50 µl EGTA (0.1 M), 37.5 µl spermidine (1 M), 15 µl spermine (0.5 M), 50 µl DTT (1 M), 250 µl PMSF (0.1 M), 500 µl sodium molybdate (1 M), 250 µl sodium vanadate (10 mM) and protease inhibitor cocktail), incubation on ice for 15 min, followed by addition of 25 µl of 10% NP 40. Vortexed samples were centrifuged (30 s, 12.000 x g, 4 °C) and incubation of the pelleted nuclei with buffer B (for 50 ml: 1 ml HEPES (1 M), 4 ml NaCl (5 M), 100 µl EDTA (0.5 M), 500 µl EGTA (0.1 M), 50 µl DTT (1 M), 250 µl PMSF (0.1 M), 500 µl sodium molybdate (1 M), 250 µl sodium vanadate (10 mM), and protease inhibitor cocktail) for 15 min at 4 °C, followed by a second centrifugation step (5 min, 12.000 x g, 4 °C). Determination of protein concentration was performed by Bradford (Bio-Rad). For Western blotting, 12% SDS polyacrylamide gels and a nitrocellulose membrane were used, and membranes were incubated with an anti-phospho-p44/42 MAP Kinase (Thr202/Tyr204), an anti-phospho-CREB (Ser133), an anti-phospho-mTOR (Ser2448), an anti-phospho-Jak1 (Tyr1034/1035), an anti-phospho-Stat3 (Ser727), an anti-phospho-Smad2 (Ser465/467), an anti-mTOR, an anti-Jak1, an anti-Stat3, an anti-Smad2, an anti-ß-actin or an anti-α-tubulin antibody (all Cell Signaling). Quantification was performed using ImageJ2 software.

### Histological analysis

Adipose tissue and liver samples were fixed in 3.7% Formafix overnight and embedded in paraffin. 1.5 μm sections (adipose tissue) and 3 μm sections (liver) were stained with hematoxylin and eosin according to standard protocols. Adipocyte size was measured in 3–6 fields at a 20x magnification.

Sections of the pancreas (1 μm) were unmasked (citrate buffer, 20 min, 92 °C), followed by staining with an anti-insulin antibody (Santa Cruz). The insulin positive areas and total pancreas surface were measured using osteomeasure software (OsteoMetrics).

Contact x-ray of formalin-fixed skeletons was performed using a Faxitron X-ray cabinet at 35 kV for 2 s. For histology of vertebral bodies (L2-L5), skeletons were fixed in 3.7% Formafix for two days, dehydrated in ascending alcohol concentrations and embedded in methylmethacrylate. 4 μm sections were stained by von Kossa/van Giesson according to standard protocols. Static histomorphometry was performed using BioQuant software (Bioquant Image Analysis Corporation).

### Cell culture

ADSCs were isolated as described in Schwabe et al., 2016 [35]. Adipocyte differentiation was induced by addition of insulin (5 µg/ml), IBMX (500 µM) and dexamethasone (1 µM) to the medium of confluent cells (α-MEM, 10% FCS, 1% penicillin/streptomycin). For Red Oil O staining, cells were fixed in 3.7% Formafix for 15 min and stained with 0.3% Red Oil O in 60% isopropanol for 2 h. For the growth curve, 4 × 10^4^ cells/well were plated in 6 well plates and over a seven-day period, trypsinized cells were counted. For stimulation experiments, cells were stimulated with dexamethasone (10 µM) or serum starved (0.5% FBS) and stimulated with insulin (5 μm/ml) or IBMX (500 µM).

Primary osteoblasts were isolated by digestion of calvariae of newborn mice as described in Jochum et al., 2000 [3]. Osteoblast differentiation was induced by addition of ß-glycerophosphate (10 mM) and ascorbate (50 µg/ml) to the medium (α-MEM, 10% FCS, 1% penicillin/streptomycin) of confluent cells. For Alizarin Red staining, cells were fixed in 90% ethanol (10 min), followed by staining with Alizarin Red (40 mM, pH 4.2, 10 min). To quantify the staining, cells were incubated with 10% acetic acid (30 min room temperature, followed by 5 min at 85 °C). After centrifugation (13.000 rpm, 10 min), ammonium hydroxide solution was added at a ratio of 1:8 and the optical density was measured at 405 nm.

### Proteomic analysis

For protein profiling pooled serum samples of at least 5 wt and *Fos*Tg mice were used. Experiments were performed by Sciomics GmbH (Heidelberg, Germany). In brief, extracted proteins were labeled with fluorescent dyes (ScioDye 1 and ScioDye 2) and the abundance of proteins was analyzed in a dual-color approach using scioDiscover antibody array. The proteins were defined as differentially expressed for ǀlog_2_FCǀ > 0.5. Pathway annotations were performed using String (https://string-db.org).

### Study design and statistical analysis

For the experiments, littermates were used as controls. Mice were assigned randomly to the groups and samples were analyzed in a blinded fashion. All data are the mean ± s.e.m. Statistical significance was determined by two-tailed Student’s t-test, Mann-Whitney-U-test, one-way ANOVA or 2-way ANOVA with Bonferroni’s comparisons test using GraphPad Prism Software (GraphPad Software Inc.). *P < 0.05, ** *P* < 0.01, *** *P* < 0.001, ****P < 0.0001.

## Results

### Decreased body weight and decreased white adipose tissue mass in Fos overexpressing mice

**Fig. 1:**
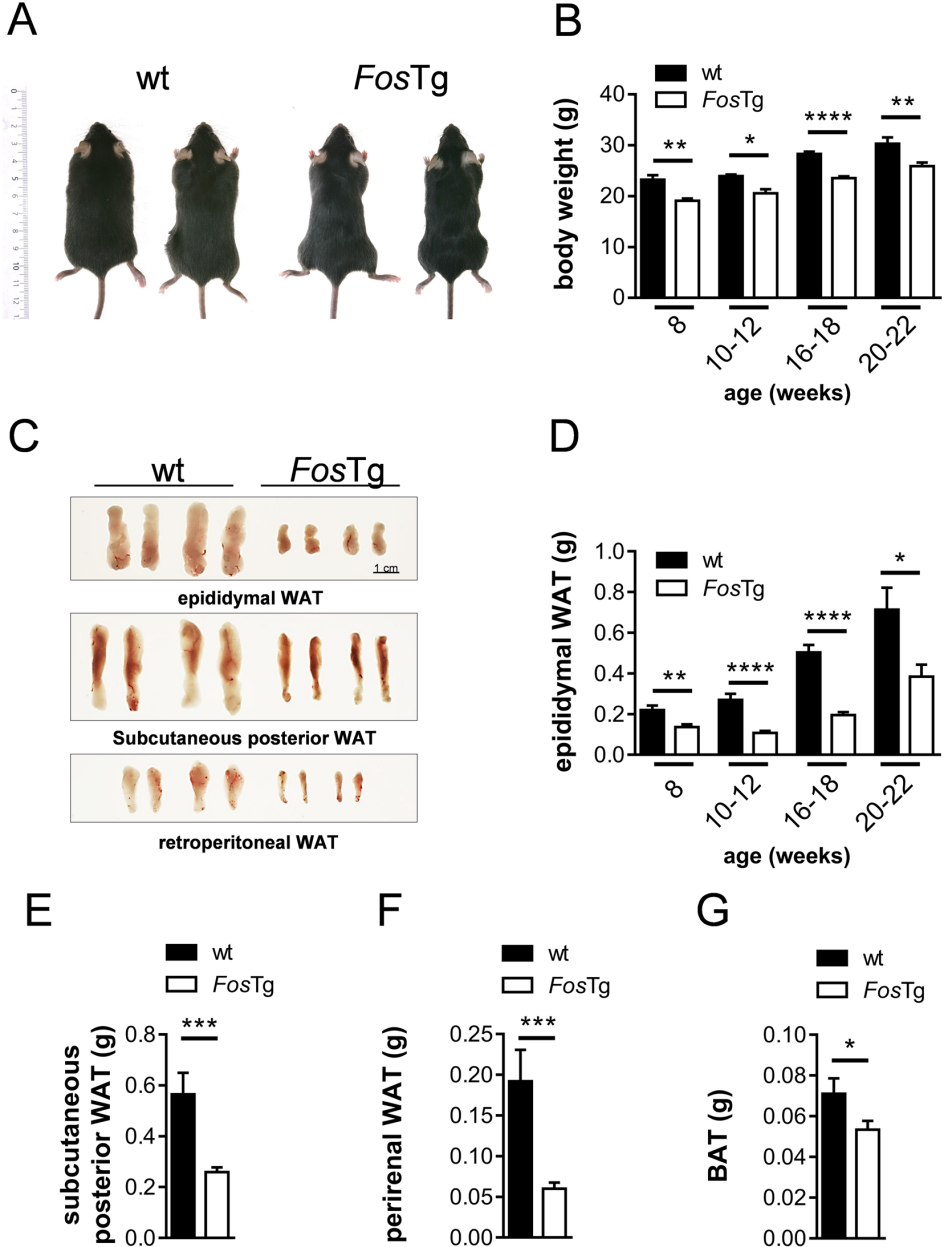
General loss of adipose tissue mass in *Fos* transgenic mice. (**A**) Representative images of 16-week-old male wild-type (wt) and *Fos *transgenic (*Fos*Tg) littermates. (**B**) Body weight of male wt and *Fos*Tg mice at the indicated ages. (**C**) Representative images of isolated adipose tissues from 16-week-old male wt and *Fos*Tg mice. (**D**) Epididymal white adipose tissue (WAT) weight of male wt and *Fos*Tg mice at the indicated ages. (**E-G**) Weight of subcutaneous posterior WAT (**E**), of the perirenal WAT (**F**) and of the brown adipose tissue (BAT) (**G**) of 16-week-old male mice. Data represent mean ± s.e.m. (*n* ≥ 4). Asterisks indicate statistically significant differences (****P < 0.0001; ***P < 0.001; **P < 0.01; *P < 0.05)

Adult *Fos* transgenic mice (thereafter termed *Fos*Tg mice) appeared smaller and leaner than their wild-type littermates (Fig. 1A). We therefore compared their age-dependent weight gain from 8 to 22 weeks of age. Decreased body weight was constantly found in the *Fos*Tg mice (Fig. 1B), suggesting decreased adipose tissue mass. To verify this, we isolated various fat pads from wild-type and transgenic mice in order to compare their size. A reduced size of all fat pads was observed in the *Fos*Tg mice (Fig. 1C and Fig. S1A). By quantifying these differences, we detected a clear decrease in the mass of all white adipose tissues (WAT) (Fig. 1D-F), as well as of brown adipose tissue (BAT) (Fig. 1G), in mice over-expressing Fos. Thus, similar to mice over-expressing Fra1 or ΔFosB [4, 36], *Fos* transgenic mice display a significantly decreased body weight associated with generally decreased adipose tissue mass.

### Fos overexpression leads to reduced adipocyte size, but does not globally affect the expression of adipocyte markers in WAT

**Fig. 2:**
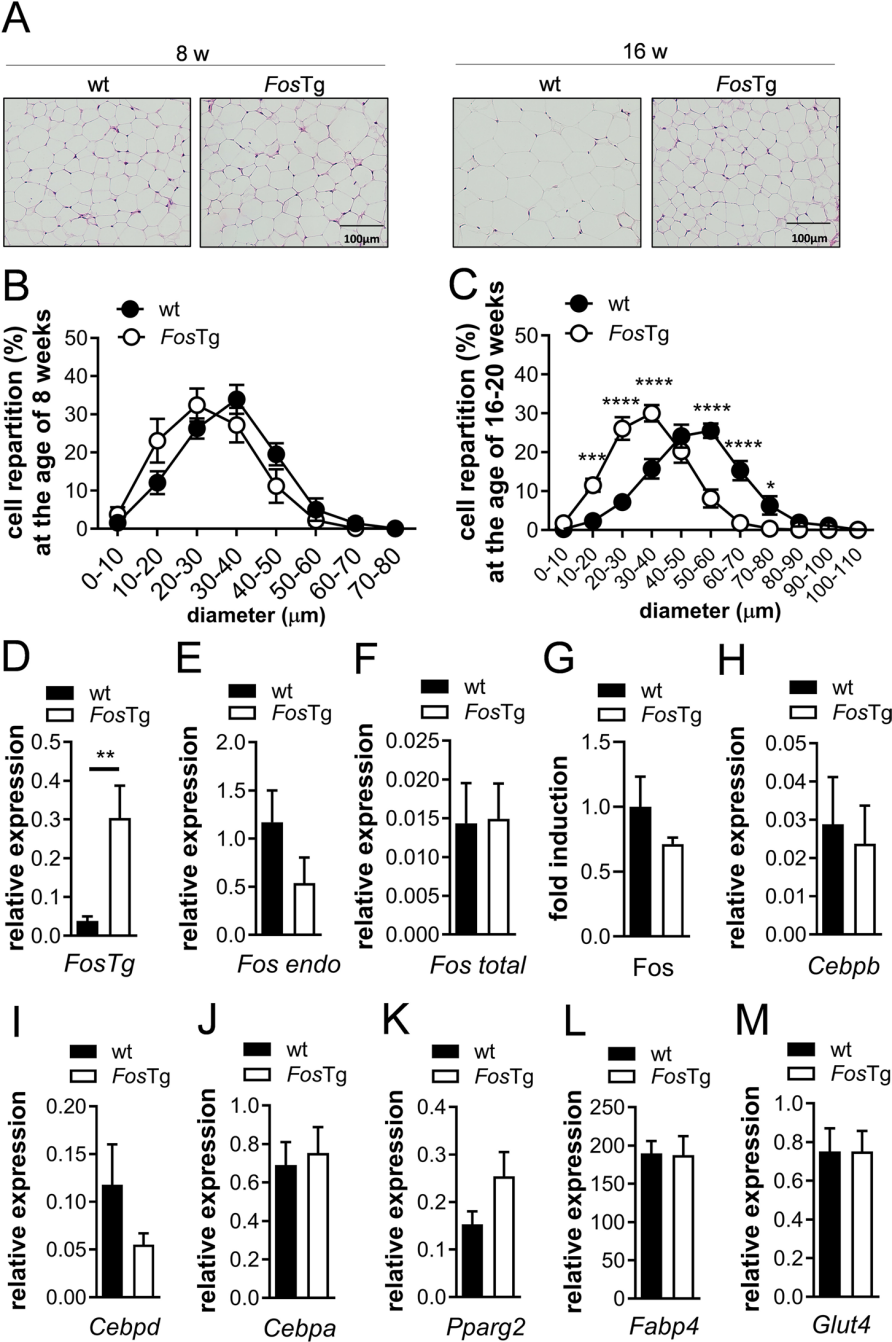
Reduced adipocyte size, but no change in the expression of adipocyte markers in WAT of *Fos*Tg mice. (**A**-**C**) Representative pictures of H&E-stained sections (**A**) and repartition curves of the adipocyte diameter of epididymal WAT from 8-week-old (**B**) and 16-20-week-old males (**C**) of the indicated genotypes. (**D**-**G**) Q-PCR analysis for expression of the *Fos* transgene (**D**), endogenous (**E**) and total *Fos* (**F**) and quantification of Fos protein levels (**G**) in epididymal WAT of 16-week-old mice. (**H-M**) Q-PCR analysis for expression of markers for white adipocytes in epididymal WAT of 16-week-old mice. Data represent mean ± s.e.m. (*n* ≥ 5). Asterisks indicate statistically significant differences (****P < 0.0001; ***P < 0.001; **P < 0.01; *P < 0.05)

We histologically analyzed the epididymal WAT of *Fos*Tg mice at the age of 8 and 16–20 weeks and performed histomorphometric quantification of the adipocyte diameter in WAT, a surrogate measurement for maturation and lipid storage of the cells. As expected, increased cell size was observed in wild-type mice during aging. However, although the size repartition of the cells of 8-week-old mice was similar in wild-type and *Fos*Tg mice, the increased cell size that was normally observed during aging was abolished in the *Fos*Tg mice (Fig. 2A-C).

The cellular phenotype observed in WAT of Fos overexpressing mice suggested that the reduced adipose tissue mass might be associated with decreased maturation of adipocytes, a phenotype reminiscent of the adipose tissue characteristics caused by overexpression of Fra1, a member of the Fos family also known to be a Fos target gene [36, 37]. Therefore, we analyzed the expression levels of Fra1 and other AP-1 members in WAT of wild-type and *Fos*Tg mice. As expected, and in agreement with its ubiquitous expression, the mRNA encoded by the transgene (*FosTg*) was found to be expressed in WAT of transgenic mice (Fig. 2D). Although expression of the transgene did not significantly affect expression of *Fosl2* (Fra2) or of any Jun family members, a significant repression of *Fosl1* (Fra1) was observed (Fig. S1B). We next performed Q-PCR analyses of endogenous and total *Fos*. We observed a non-significantly reduced expression of endogenous *Fos* in *Fos*Tg WAT, leading to similar total *Fos* expression levels in wild-type and *Fos*Tg mice (Fig. 2E-F). The absence of increased Fos expression was confirmed at the protein level (Fig. 2G). Nevertheless, to exclude a potential cell-autonomous mechanism explaining the fat phenotype of *Fos*Tg mice, we performed Q-PCR analyses for adipocyte differentiation and activity markers. No clear reduction was found when quantifying the expression of transcription factors regulating early differentiation (*Cebpb* and *Cebpd*) or maturation (*Cebpa and Pparg*) of adipocytes (Fig. 2H-K). Markers characterizing differentiated adipocytes (*Fabp4* and *Glut4*) were also unaffected (Fig. 2L-M). These findings suggested that white adipocyte differentiation and function are not globally affected in our *Fos* transgenic model.

### The Fos-induced adipose tissue phenotype is not cell-autonomous

We isolated adipose-derived stem cells (ADSCs) from adult mice to further omit the potential cell-autonomous effect of Fos overexpression on white adipocyte differentiation in vitro. First, we established cellular growth curves that did not reveal any difference, thus excluding a proliferation or apoptosis phenotype (Fig. S2A). We next compared the response to adipogenic stimulation of the ADSCs isolated from wild-type and *Fos*Tg mice. Here, we first studied the response of the cells to IBMX by analyzing the phosphorylation of Creb. A comparable level of Creb phosphorylation was observed using Western blotting following IBMX stimulation (Fig. S2B). Similarly, we demonstrated the functionality of the insulin receptor in ADSCs isolated from wild-type and *Fos*Tg mice by monitoring the activation of ERK in response to short term stimulation by insulin. A similar level and kinetic of ERK phosphorylation were observed by Western blot (Fig. S2C). Finally, a similarly increased expression of *Gilz1* and *Per1*, two typical glucocorticoid target genes, were observed after dexamethasone treatment, thereby demonstrating a normal response of adipocyte progenitor cells to glucocorticoids (Fig. S2D and E). Thus, ADSCs isolated from *Fos*Tg mice are normally responsive to adipogenic stimuli in vitro and are therefore expected to normally differentiate into adipocytes.

**Fig. 3:**
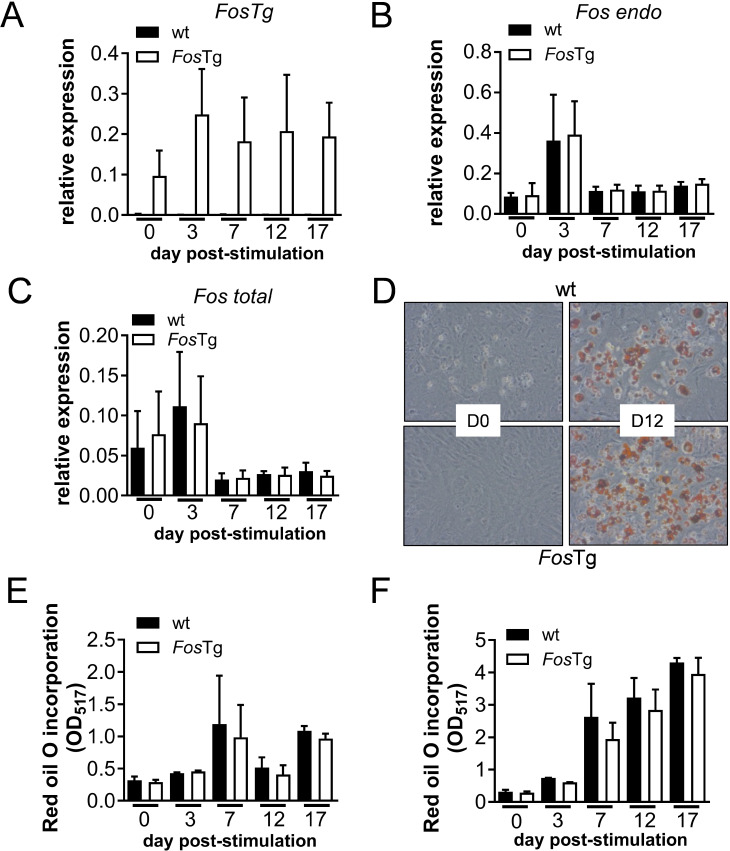
The fat phenotype of *Fos* transgenic mice is not caused by a cell-autonomous defect in adipocyte differentiation. (**A**-**C**) Q-PCR analysis for expression of the *Fos* transgene (**A**), endogenous *Fos* (**B**) and total *Fos* (**C**) in wt and *Fos*Tg ADSCs during the course of adipocyte differentiation. (**D**) Representative images of wt and *Fos*Tg ADSCs after Red oil O staining (in red) at day 0 (confluency) or 12 days after inducing the adipocyte differentiation. (**E**) Quantitative analysis of the Red oil O staining of ADSCs isolated from subcutaneous WAT of wild-type (wt) and *Fos*Tg mice at different time points in cultures without adipogenic stimulation. (**F**) Quantitative analysis of the Red oil O staining at different time points in cultures with adipogenic stimulation. Data represent mean + s.e.m. (n ≥ 3 independent cell isolations)

To test this hypothesis, we next induced adipogenesis by treating confluent cells with insulin, IBMX and dexamethasone. The transgene was expressed in cells isolated from *Fos*Tg mice and its level of expression was not affected by adipogenic stimulation (Fig. 3A). We next analyzed endogenous and total *Fos* expression levels. Endogenous *Fos* displayed a transient peak at day 3 of adipogenic differentiation in both genotypes, indicating the importance of Fos in early adipogenesis (Fig. 3B). However, total *Fos* expression levels were similar in wild-type and *Fos*Tg mice (Fig. 3C). To prove the absence of an effect mediated by the transgene, we performed Red Oil O staining to demonstrate the efficacy of adipocyte differentiation and did not detect any qualitative difference between cultures from wild-type and *Fos*Tg mice (Fig. 3D). This observation was confirmed by quantitative analysis, which demonstrated that there was no difference in the spontaneous adipocyte differentiation in the absence of stimulation (Fig. 3E). Importantly, the cells responded with a similar kinetic and amplitude to the adipogenic stimulation (Fig. 3F). These data were further verified by Q-PCR analysis for expression of adipocyte markers, including *Pparg2*, *Cebpa*, *Glut4* and *Fabp4*, as well as adipokines which were all unaffected by expression of the transgene (Fig. S3A to H).

As ADSCs are adipose tissue resident mesenchymal stem cells with the capacity to differentiate into osteoblasts [38], we also performed osteogenic differentiation of the ADSCs, which demonstrated the normal capacity of cells isolated from the *Fos*Tg mice to mineralize in response to stimulation with β-glycero-phosphate and ascorbic acid (Fig. S4A and B). Likewise, no change was observed when quantifying bone parameters in tumor-free areas of bones from *Fos*Tg mice (Fig. S4C and D). Thus, the decreased WAT mass in *Fos*Tg mice is independent of a cell-autonomous defect affecting mesenchymal progenitors and thus adipogenesis, as well as of a switch from adipogenesis to osteoblastogenesis.

### Decreased circulating glucose but normal insulin levels in *Fos*Tg mice

**Fig. 4:**
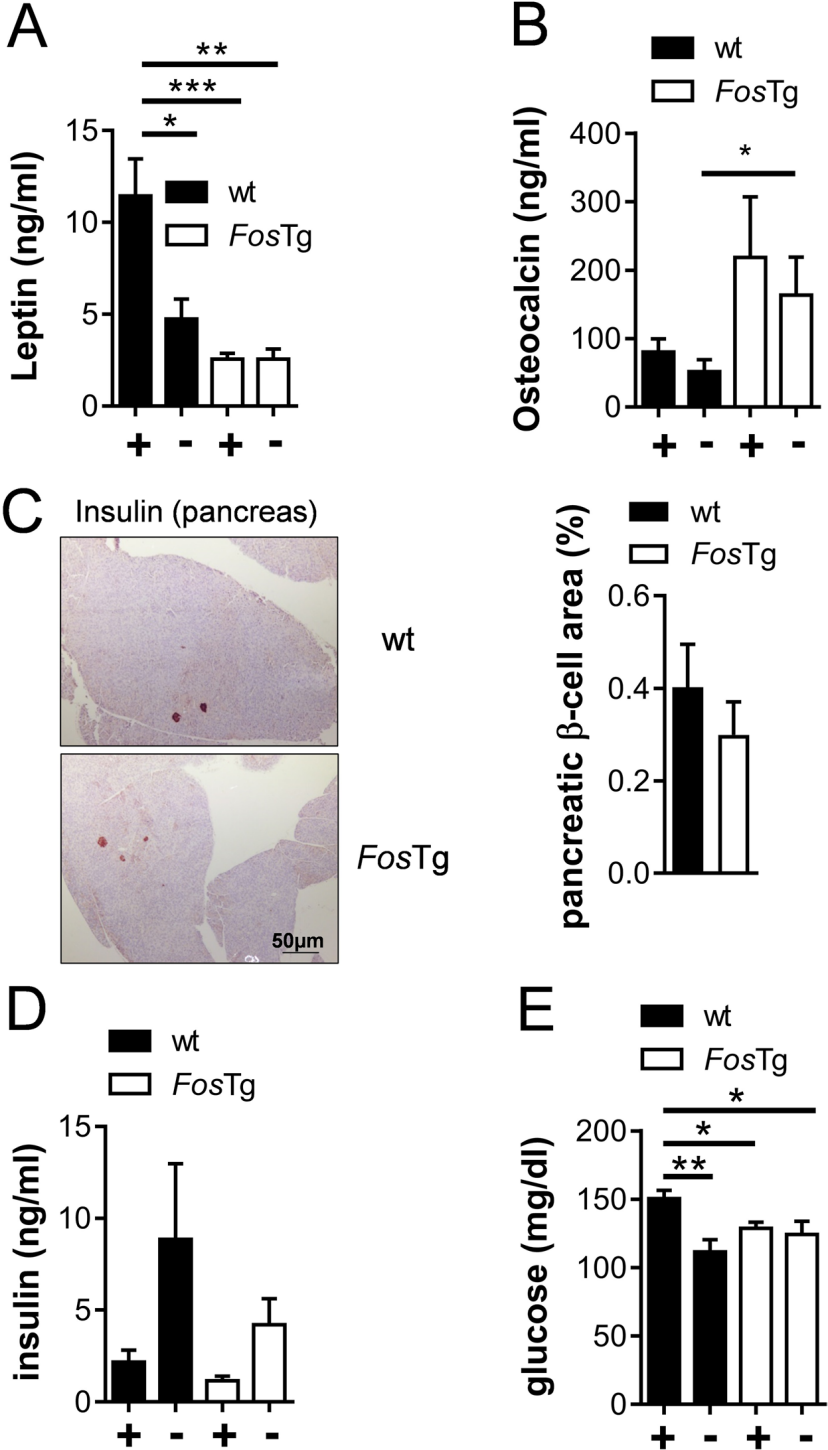
Decreased serum glucose levels in *Fos*Tg mice. (**A**) ELISA-based quantification of circulating leptin concentrations in 16-week-old mice fed (+) or starved for 6 h (-). (**B**) Circulating levels of total osteocalcin. (**C**) Representative images showing immunostaining for insulin in the pancreas of 16-week-old mice and quantification of the β-cell area. (**D**-**E**) Circulating levels of insulin (**D**) and glucose (**E**) in fed (+) and starved (-) mice. Data represent mean ± s.e.m. (*n* ≥ 5). Asterisks indicate statistically significant differences (***P < 0.001; **P < 0.01; *P < 0.05)

Since the fat phenotype in *Fos*Tg mice was not explained by a cell-autonomous defect in adipogenesis, we analyzed for potential variations in the systemic regulation of adipose tissues related to the known interaction of osteoblasts and adipocytes. We therefore measured the circulating levels of hormones that have been shown to couple bone and adipose tissue metabolism in 16-week-old mice. In agreement with their lean phenotype, leptin, a hormone produced by mature adipocytes to control satiety, was decreased in the *Fos*Tg mice and was not sensitive to food deprivation (Fig. 4A). In contrast, levels of osteocalcin, a hormone produced by osteoblasts, were found increased in sera of *Fos*Tg mice (Fig. 4B), probably as a result of its production by the tumors. As osteocalcin has been shown to induce the production of insulin via an increased proliferation of pancreatic β-cells, we performed insulin immunostaining in order to quantify the average volume of β-cell islets on pancreas sections, a commonly used surrogate method to evaluate proliferation of these cells. No difference in the average surface occupied by insulin-positive cells was observed when comparing the two genotypes (Fig. 4C). Likewise, no increase in the circulating levels of insulin could be detected and a physiologically increased insulin secretion was observed in the blood of *Fos*Tg mice following food deprivation (Fig. 4D). However, serum glucose levels were significantly lower in the *Fos*Tg mice after feeding, and the expected lowering of glucose was not observed after food deprivation (Fig. 4E). However, the expression level of the adipokines resistin and Il6, that were examined in this context, were not affected in white adipose tissue (Fig. S5A-B).

### Increased serum Fgf21 concentrations in *Fos* transgenic mice

**Fig. 5:**
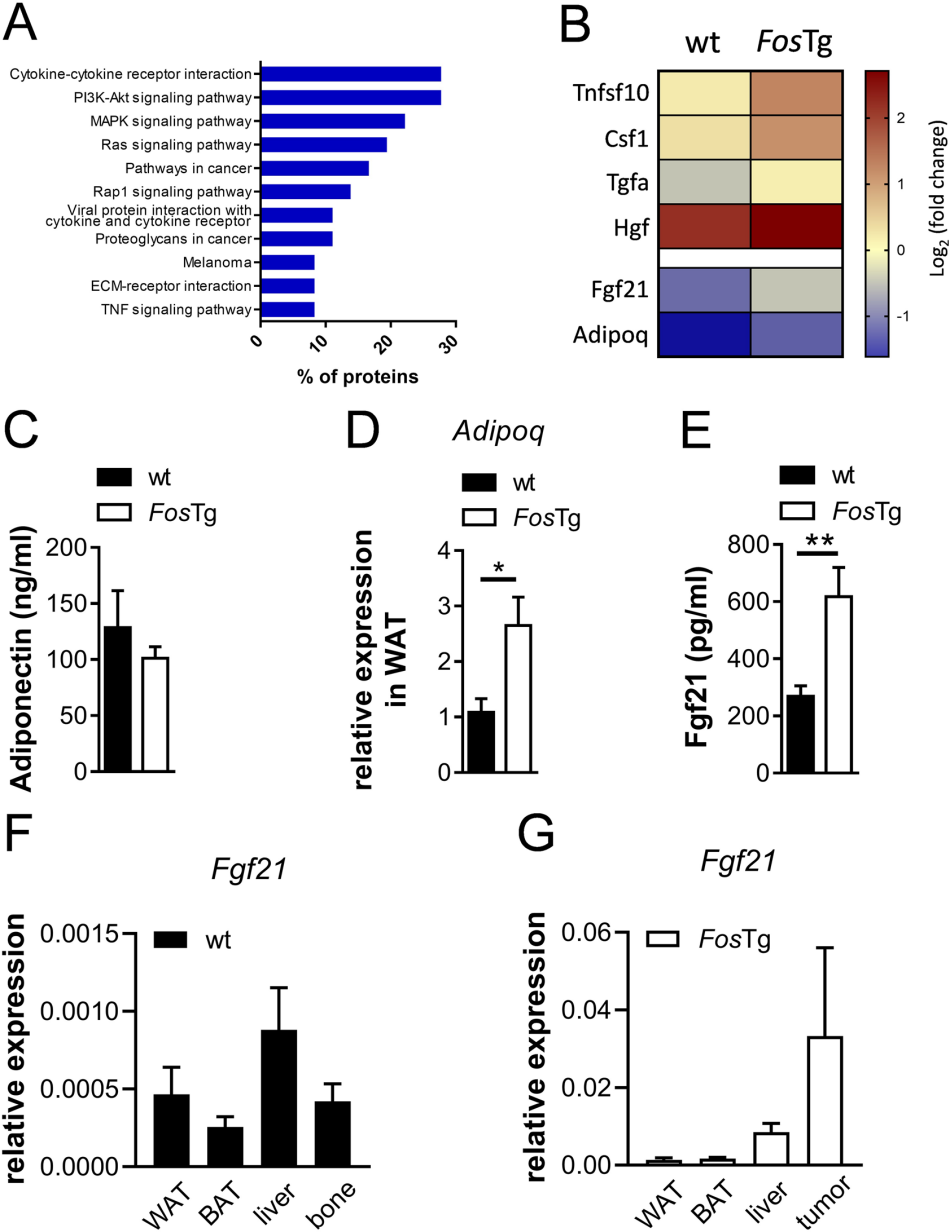
Increased serum Fgf21 concentrations in *Fos* transgenic mice. (**A**-**B**) Results of the antibody microarray analysis: Selection of most affected pathways showing the percentage of proteins within each pathway (**A**). Heat map of differentially expressed secreted factors related to cachexia in wt compared to *Fos*Tg mice (**B**). (**C**) ELISA-based quantification of circulating adiponectin concentrations. (**D**) Quantitative Real-time PCR analysis of *Adipoq* expression in the white adipose tissue of 16-week-old mice. (**E**) Circulating levels of Fgf21 in wt compared to *Fos*Tg mice. (**F**-**G**) qRT-PCR analyses of *Fgf21* expression in white and brown adipose tissue, liver and bone samples of wild-type mice (**F**), and of white and brown adipose tissue, liver and tumor samples in *Fos*Tg mice (**G**). Data represent mean ± s.e.m. (*n* ≥ 3). Asterisks indicate statistically significant differences (**P < 0.01; *P < 0.05)

To assess the mechanism responsible for the metabolic phenotype of *Fos*Tg mice, we employed a proteomic approach. Thereby, the abundance of 1400 proteins was determined in pooled murine serum samples of wt and *Fos*Tg mice. Functional annotation of factors using STRING showed pathways related to cancer and TNF signaling, as well as PI3K-Akt/MAPK/Ras/Rap1 signaling as main pathways affected when focusing on differentially expressed secreted factors in the osteosarcoma-developing *Fos*Tg mice compared to control (Fig. 5A). By analyzing this dataset, we found an increased abundance of cachexia-inducing factors, namely Tnfsf10, Csf1, Tgfa and Hgf in *Fos*Tg mice. Furthermore, the analysis revealed an increased level of Fgf21, a factor described to be elevated in patients with cachexia [39], and of its target gene adiponectin in the sera of *Fos*Tg mice (Fig. 5B). To confirm the results of the antibody microarray analysis, we applied ELISA and qRT-PCR. Despite an increased expression of adiponectin in adipose tissue on RNA level, its serum levels were not significantly elevated (Fig. 5C-D). Nevertheless, we observed increased concentrations of Fgf21 in the serum of *Fos*Tg mice (Fig. 5E). To address the tissue specificity of *Fgf21* expression we compared the RNA expression levels in white and brown adipose tissue, liver and bone/tumor of wild-type and *Fos*Tg mice. In wild-type mice, *Fgf21* showed the highest expression in the liver, which is described as primary site of *Fgf21* production (Fig. 5F). In *Fos*Tg mice, *Fgf21* RNA levels were significantly elevated in BAT, liver and tumor with high expression levels in liver and tumor tissue, emphasizing the importance of these organs for *Fgf21* expression (Fig. S6A-D and Fig. 5G).

### Increased insulin sensitivity in *Fos*Tg mice

**Fig. 6:**
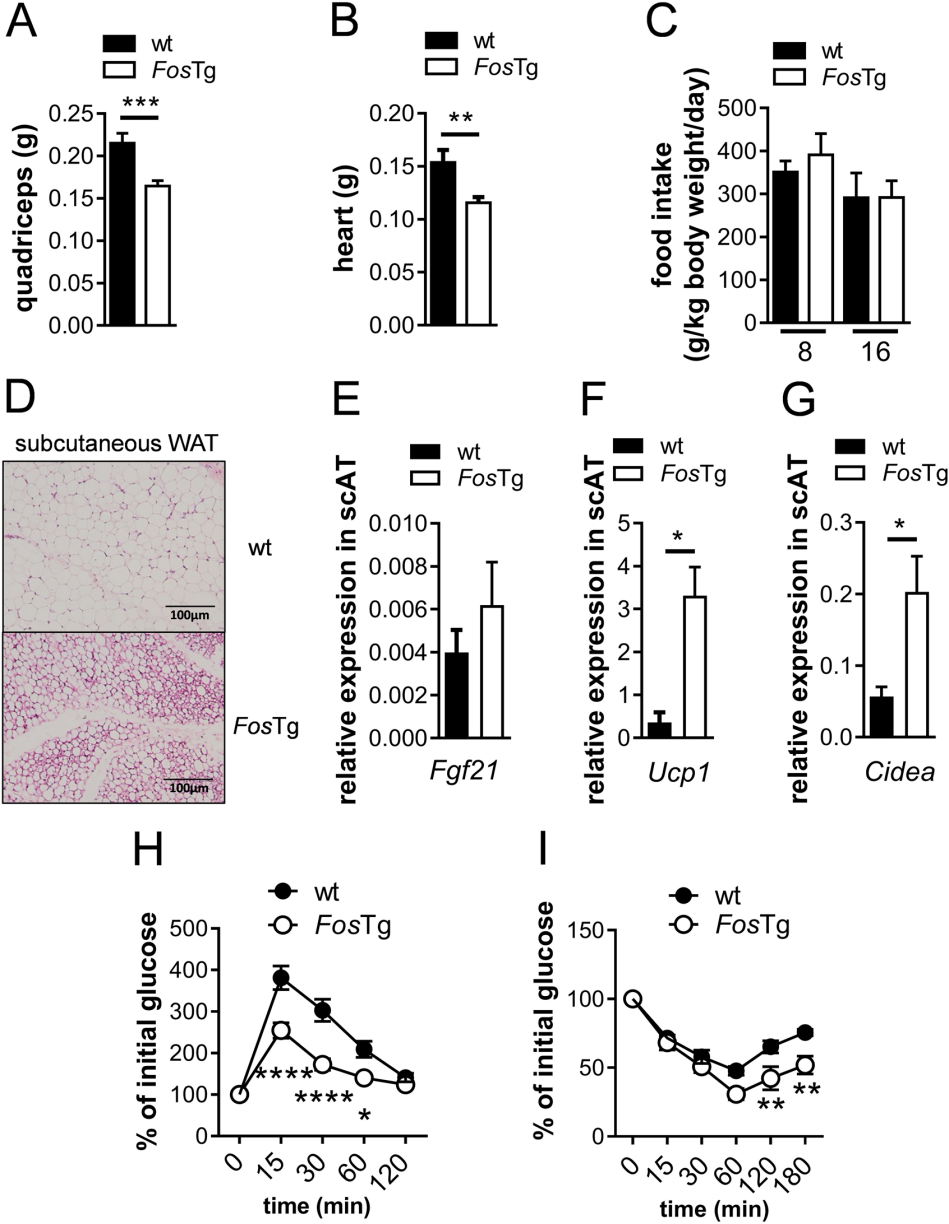
Increased glucose tolerance and insulin sensitivity in *Fos*Tg mice. (**A**-**B**) Quadriceps weight (**A**) and heart weight (**B**) of 16-week-old male wt and *Fos*Tg littermates. (**C**) Quantification of food intake in 8- and 16-week-old wt and *Fos*Tg mice. (**D**) Representative pictures of H&E stained sections of the subcutaneous white adipose tissue from 16-week-old males of the indicated genotype. (**E**-**G**) Q-PCR analysis for expression of *Fgf21* (**E**) and *Ucp1* (**F**) and *Cidea* (**G**) in the subcutaneous adipose tissue of *Fos*Tg mice relative to wt. (**H**) Glucose tolerance test in 16-week-old wt and *Fos*Tg mice. (**I**) Insulin tolerance test in 16-week-old wt and *Fos*Tg mice. Data represent mean ± s.e.m. (*n* ≥ 4). Asterisks indicate statistically significant differences (****P < 0.0001; ***P < 0.001; **P < 0.01; *P < 0.05)

Cachexia is characterized by a reduced muscle mass [40]. Furthermore, Fgf21, that was shown to induce skeletal muscle atrophy, has been described to play a role in cachexia [41, 42]. Therefore, we measured the weight of the quadriceps, which was found to be reduced in *Fos*Tg mice (Fig. 6A). Similarly, the heart weight was decreased in mice overexpressing Fos compared to control (Fig. 6B). However, the reduction in muscle mass as well as of the total body weight of *Fos* transgenic mice was not caused by anorexia, as we could exclude a decreased food consumption by comparing food intake in wild-type and *Fos*Tg mice at the ages of 8 and 16 weeks (Fig. 6C).

As in cachexia, an increase of pro-inflammatory cytokines in the liver is typically described, we performed Q-PCR analyses of *Tnfa*, *Il1b*, *Il6* and *S100a9* expression. These genes seem to be elevated in the liver of *Fos*Tg mice, with *Tnfa* and *Il1b* being significantly increased (Fig. S7A-D).

Next, we analyzed the subcutaneous white adipose tissue of *Fos*Tg mice for symptoms of cachexia. Histological examination of the subcutaneous white adipose tissue, which is the typical site of adipocyte browning, showed, similar to the gonadal adipose tissue, adipocytes with a reduced cell size (Fig. 6D). Importantly, while *Fgf21* expression is not significantly elevated, we observed an increased expression level of the thermogenic gene *Ucp1* and *Cidea*, markers that are associated with WAT browning, leading to the increased energy expenditure observed in cachexia (Fig. 6E-G).

As Fgf21 was described to regulate insulin sensitivity [27], glucose and insulin tolerance tests were performed. Here, *Fos*Tg mice showed an increased rate of glucose clearance compared to wild-type controls (Fig. 6H). Furthermore, Fos overexpression was found to enhance insulin sensitivity (Fig. 6I).

Taken together, *Fos*Tg mice show multiple symptoms of cachexia, that have been described to be regulated via Fgf21, namely reduced muscle mass, signs of browning of subcutaneous white adipose tissue and increased insulin sensitivity.

### Decreased lipogenesis in the liver of *Fos*Tg mice

**Fig. 7:**
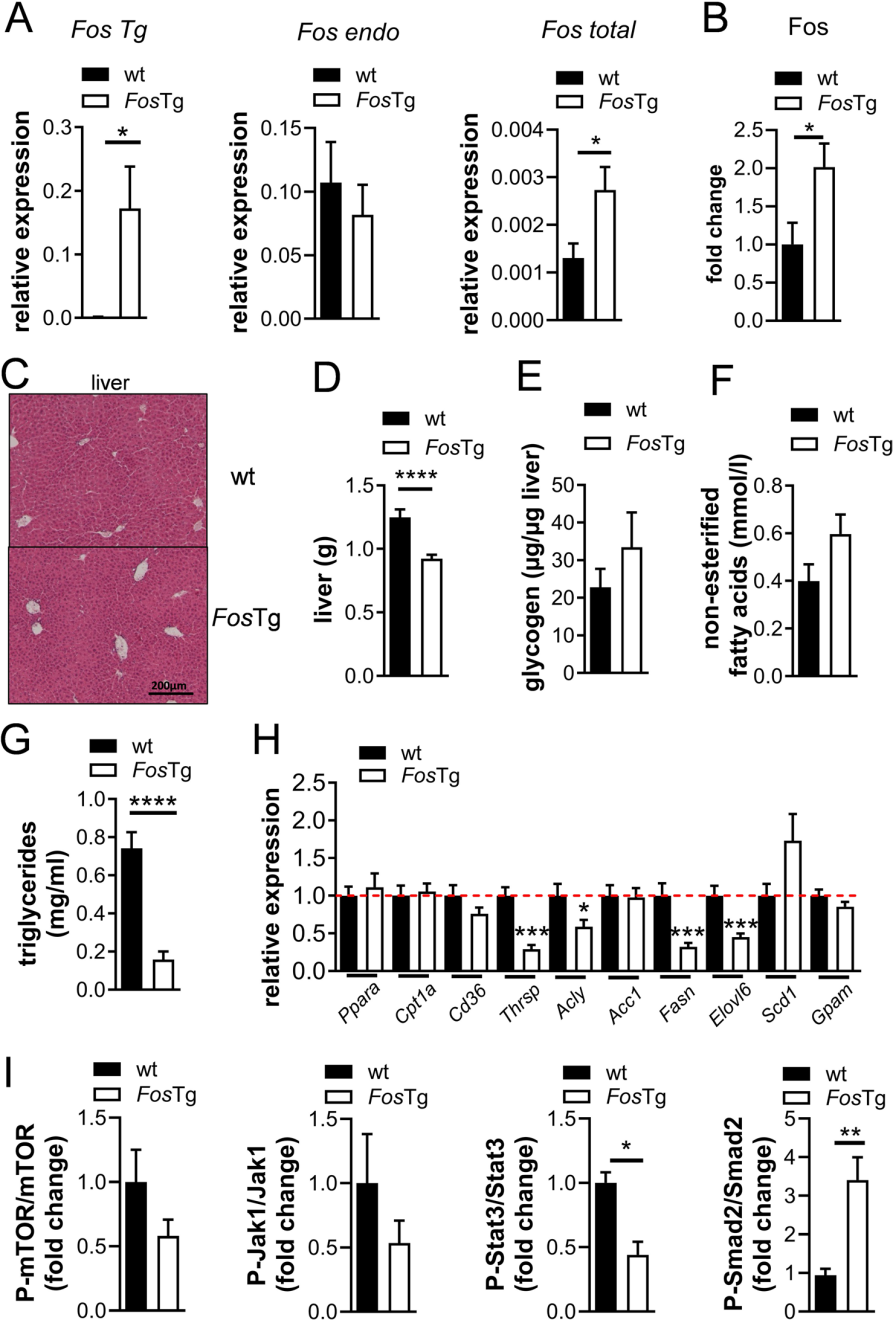
Impaired lipogenesis in the liver of *Fos* transgenic mice. (**A**) Q-PCR analysis for expression of the *Fos* transgene, endogenous *Fos* and total *Fos* in the liver of 16-week-old wt and *Fos*Tg mice. (**B**) Quantification of Fos protein levels in the liver of *Fos*Tg mice compared to wild-type. (**C**) Representative pictures of H&E stained sections of the liver from 16-week-old males of the indicated genotype. (**D**) Liver weight of wt and *Fos*Tg mice. (**E**) Glycogen content in the liver of 16-week-old wt and *Fos*Tg males. (**F**) Serum levels of non-esterified fatty acid and (**G**) triglycerides in the serum of 16-week-old wt and *Fos*Tg mice. (**H**) Q-PCR analysis for expression of genes controlling lipogenesis in the liver. (**I**) Quantification of Western Blot analyses for mTor, Jak1, Stat3 and Smad2 phosphorylation in liver samples of wt and *Fos*Tg mice. Tubulin detection was used as loading control. Data represent mean ± s.e.m. (*n* ≥ 6). Asterisks indicate statistically significant differences (****P < 0.0001; ***P < 0.001; **P < 0.01; *P < 0.05)

Fgf21 was further shown to decrease lipogenesis and to increase gluconeogenesis and fatty acid oxidation in the liver [43]. Therefore, we evaluated the glucose and lipid metabolism in the liver of *Fos*Tg mice compared to control. We confirmed Fos transgene expression in the liver of transgenic mice (Fig. 7A). While endogenous *Fos* levels remained unchanged, total *Fos* expression was significantly higher in *Fos*Tg mice compared to wild-type animals as demonstrated at RNA and at protein level (Fig. 7A-B and Fig. S8A). Despite the observed adipose tissue phenotype, no histological evidence of liver steatosis was observed (Fig. 7C-D). In addition, we did not observe a significant difference in the glycogen content in the liver despite tumor formation in *Fos*Tg mice (Fig. 7E). Next, we analyzed the serum level of non-esterified fatty acids and triglycerides. In accordance with the effects of Fgf21 in the liver, the level of non-esterified fatty acids was not affected, while the level of triglycerides was strongly reduced in *Fos*Tg compared to control mice (Fig. 7F-G). Transcriptional regulators of enzymes involved in ß-oxidation (*Ppara* and *Cpt1a*) were not differentially expressed in *Fos*Tg mice (Fig. 7H). Importantly however, several enzymes regulating lipogenesis and elongation of long-chain fatty acids, namely *Thrsp*, *Acly*, *Fasn* and *Elovl6* showed significantly decreased expression levels (Fig. 7H and Fig. S8B).

Mechanistically, we analyzed the activation of the Jak-Stat-pathway that is induced by Il6, for which the circulating levels correlate with the development of cachexia [44]. We neither observed an increased phosphorylation of Jak1 nor of mTor. In addition, the Stat3 phosphorylation levels were significantly decreased, possibly reflecting a compensatory effect of Fgf21. However, Smad2, which is activated by Tgf-ß, showed increased phosphorylation in *Fos*Tg liver samples compared to control samples (Fig. 7I and Fig. S8C).

Taken together, these data strongly suggest that the reduced adipose tissue mass in *Fos*Tg mice can be explained by increased serum concentrations of Fgf21, leading to reduced lipogenesis in the liver.

### Reducing Fos-induced tumor burden leads to reduced Fgf21 levels and an ameliorated adipose tissue phenotype

**Fig. 8:**
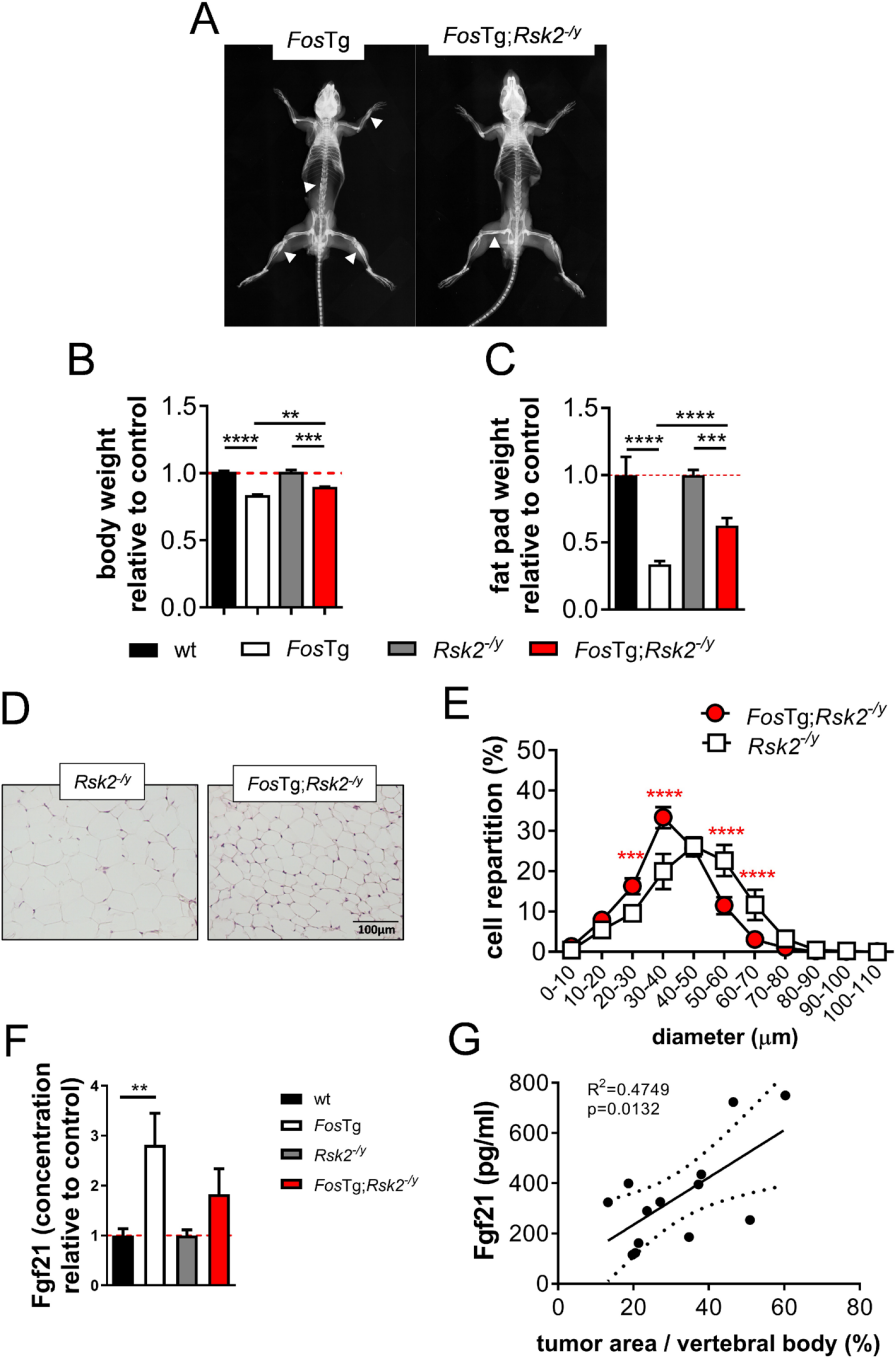
The adipose tissue phenotype of *Fos* transgenic mice is caused by osteosarcoma formation. (**A**) Representative X-rays of 16-week-old *Fos*Tg and Rsk2-deficient *Fos*Tg males (*Fos*Tg;*Rsk2*^*−/y*^). (**B**) Body weight of 16-week-old *Fos*Tg and *Fos*Tg;*Rsk2*^*−/y*^ males relative to the respective parental line (wt or *Rsk2*^*−/y*^). (**C**) Epididymal white adipose tissue (WAT) weight relative to the respective parental line in the same mice. (**D**) Representative pictures of H&E-stained sections of epididymal WAT from 16-week-old males of the indicated genotypes. (**E**) Repartition curves of the adipocyte diameter in epididymal WAT of 16 to 20-week-old males of the indicated genotype. (**F**) Elisa-based quantification of serum Fgf21 levels in *Fos*Tg and *Fos*Tg;*Rsk2*^*−/y*^ relative to the respective parental line (wt or *Rsk2*^*−/y*^). (**G**) Correlation between serum Fgf21 levels and tumor area per vertebral body of *Fos*Tg mice. Data represent mean ± s.e.m. (*n* ≥ 6). Asterisks indicate statistically significant differences (****P < 0.0001; ***P < 0.001; **P < 0.01; *P < 0.05)

To determine whether the metabolic phenotype developing in *Fos*Tg mice is a consequence of tumor growth, we genetically inactivated the Ribosomal S6 kinase (Rsk2) in *Fos* transgenic mice (*Fos*Tg;*Rsk2*^*−/y*^ mouse line) in order to reduce tumor development [32]. First, we confirmed our original observation that inactivating Rsk2 in *Fos*Tg mice strongly decreases tumor growth (Fig. 8A). We next compared the weight parameters of *Fos*Tg and *Fos*Tg;*Rsk2*^*−/y*^ mice to the two parental lines (*wt* and *Rsk2*^*−/y*^), respectively. At the age of 16 weeks, body weight of *Fos*Tg;*Rsk2*^*−/y*^ animals was less decreased than the body weight of *Fos*Tg mice compared to the respective controls (Fig. 8B). Likewise, the fat pad weight was also reduced in *Fos*Tg mice lacking Rsk2, however significantly less pronounced than in *Fos*Tg mice compared to wt (Fig. 8C). Histological analyses of the epididymal WAT of *Rsk2*^*−/y*^ compared to *Fos*Tg;*Rsk2*^*−/y*^ mice at the age of 16 weeks suggested a reduced cell size in the white adipose tissue of *Fos*Tg;*Rsk2*^*−/y*^ mice (Fig. 8D). Quantification showed, that while the repartition curve of the adipocyte diameter in the *Rsk2*^*−/y*^ animals was comparable to wild-type mice, a decreased cell size was also evident in the *Fos*Tg;*Rsk2*^*−/y*^ mice, that was, nevertheless, less pronounced than in *Fos*Tg mice (Fig. 8E). Thus, the fat phenotype observed in the *Fos* transgenic mice is associated with the tumor growth, thereby suggesting that the metabolic phenotype observed in these mice is linked to tumor cachexia.

Next, we measured the concentration of circulating Fgf21. In accordance with the association of reduced adipose tissue mass and tumor burden, Fgf21 serum levels are less increased in *Fos*Tg; *Rsk2*^*−/y*^ mice than in *Fos*Tg mice compared to the respective control (*Rsk2*^*−/y*^ mice and wt mice, respectively) (Fig. 8F). In contrast, Tnfa, that was reported to be regulated by Fos [45] and potentially responsible for transmitting the effect, was not affected (Fig. S9). Finally, we plotted Fgf21 serum levels of *Fos*Tg mice to the tumor area in the vertebral bodies of the same mice. Importantly, we observed a direct correlation between serum Fgf21 levels and tumor burden in the respective mice (Fig. 8G).

Therefore, these data uncover a new phenotype of transgenic mice over-expressing Fos, that in addition to osteosarcoma development show a reduced adipose tissue mass but also develop an increased glucose tolerance and insulin sensitivity that can be explained by an increased concentration of circulating Fgf21. Most importantly, serum levels of Fgf21 are correlating with tumor burden in *Fos*Tg mice.

## Discussion

The analyses of loss- and gain-of-function of AP-1 transcription factor family members in mice showed not only the important role of AP-1 in the regulation of bone mass and cancer development, but also as cell-autonomous and/or systemic regulators of fat metabolism and adipose tissue mass [4, 36, 46–49]. In this publication, we show that a further member of the AP-1 transcription factor family, namely Fos, that, when overexpressed, causes osteosarcoma formation [7], is regulating lipid metabolism and insulin tolerance. Mechanistically, we found an elevated Fgf21 level in the serum of *Fos*Tg mice compared to control animals that can explain the phenotype of reduced lipogenesis in the liver and a reduced fat content in *Fos*Tg mice. 

*Fos* transgenic mice show a reduced white adipose tissue mass during aging associated with a reduced adipocyte size. This adipose tissue phenotype does not seem to be caused by a cell-autonomous mechanism, unlike what was observed in mice overexpressing *Fra1* [36]. A cell-autonomous effect is also contributing to the phenotype described for mice with an adipocyte-specific deletion of *Fra2* as well as for *DeltaFosB* overexpressing mice [47, 48]. The non-cell-autonomous nature of the fat phenotype in *Fos*Tg mice is supported by several arguments: First, the expression levels of adipocyte differentiation and maturation markers were unchanged in adipose tissue of *Fos*Tg mice. Second, overexpression of Fos did not block the differentiation capacity of adipose derived stem cells in vitro. And third, common signaling pathways and gene induction were not affected in stimulation experiments.

In our study, we analyzed H2kb-*Fos*Tg mice, a mouse model where Fos is ubiquitously over-expressed under the H2Kb promoter [7, 30], including expression in white adipose tissue, but also in liver. A further site of transgene overexpression is bone tissue, leading to spontaneous osteosarcoma formation [50]. Given the ubiquitous over-expression of Fos, we performed serum antibody microarray analyses where we found Fgf21 levels to be increased, a result that we confirmed by ELISA as well as on RNA level. The described effects of increased Fgf21 concentrations, namely improved insulin sensitivity and glucose clearance as well as decreased lipogenesis in the liver, mirrored the phenotype of *Fos*Tg mice.

Fgf21 expression in the white adipose tissue was described to be regulated by Pparg, a transcription factor activated by the AP-1 member Fos [51, 52]. While we found Pparg expression to be slightly elevated in white adipose tissue, and therefore cannot exclude the Ppar signaling pathway to mediate the metabolic phenotype of *Fos*Tg mice, we could not observe an increase in the RNA level of Ppara in the liver, that is known to regulate Fgf21 expression in this tissue [53]. Therefore, we suppose a Ppar-independent regulation of Fgf21 by Fos. Indeed, a direct regulation of Fgf21 expression by Fos can be suggested, given that Xiao et al. also showed the occurrence of an AP-1 binding site in the promoter of Fgf21, that is bound by the AP-1 member cJun, thereby regulating adipose tissue mass by directly controlling Fgf21 expression [54]. Furthermore, Zhang et al. demonstrated in vitro that AP-1 activation is required for the chemotherapeutic drug cisplatin to induce Fgf21 expression and that TPA, a phorbol ester that activates AP-1 signaling, can induce Fgf21 expression, effects that are normalized by pre-treatment with an AP-1 inhibitor [55]. However, if the AP-1 member Fos directly induces the expression of Fgf21 and therefore, whether the relationship between Fos and Fgf21 is causal or correlational has not yet been described and has to be further investigated.

Furthermore, in the *Fos*Tg mouse model, we observe a phenotype resembling the cachexia syndrome, which can develop in advanced stages of cancer. The state of chronic inflammation in cachexia, leads, for example via proinflammatory cytokines like Il6, to an activation of the Jak/Stat3 signaling pathway, causing muscle atrophy [56, 57]. Inhibitors targeting Jak1/2 have furthermore been described to block adipocyte lipolysis in cachexia [58]. In addition, Fgf21 was described to be regulated via Jak/Stat signaling by different cytokines [59, 60]. TNFa was additionally described to play a crucial role in cachexia and for the AP-1 member JunB, it has been demonstrated that adipose-specific deletion enhances thermogenesis [61] and that JunB-ko mice show a reduced adipose tissue mass that was explained by an increased lipolytic rate accompanied by an increased level of TNFa [46]. Increased Il6 levels could not be detected by proteomic analysis, and even though we observed an elevated level of *Pnpla2*, an enzyme catalyzing the first step of hydrolysis of triglycerides, in the white adipose tissue of *Fos*Tg mice (data not shown), Tnfa, while increased on RNA level in the liver, was not elevated in the serum of *Fos*Tg mice. Still, we observe a reduced phosphorylation of Stat3, possibly caused by a feedback mechanism of Fgf21 [62]. However, we found several additional cachexia-inducing factors, possibly responsible for the induction of the cachexia-like phenotype, to be upregulated in *Fos*Tg mice. Another aspect to consider is that AP-1 action has been shown to depend on the dimer composition [63, 64], and it was described that the combination of AP-1 family members is differentially regulating lipid metabolism [51]. Thus, we suppose that the composition of the AP-1 dimer can systemically regulate fat metabolism and further research would be needed to establish the specific function of Fos in the context of cachexia and Fgf21 induction.

The main limitation of our study is, that with our analyses we cannot exclude that a factor different from Fgf21 may influence fat mass and liver metabolism in *Fos*Tg mice. Therefore, a follow-up study should aim to directly test whether Fgf21 mediates the observed adipose tissue and liver phenotypes using *Fgf21* knock-out studies or inhibitor treatments. Furthermore, Fgf21 has been associated with improved adipocyte function, systemic glucose and lipid clearance and energy expenditure. Therefore, we cannot exclude that Fos exerts its effects on adipose tissue via systemic metabolic alterations independent of the cachexic phenotype and a more detailed assessment of lipid and glucose metabolism should be performed in a model overexpressing Fos specifically in adipocytes. It is also important to state that, as metabolic alterations are caused by tumor development, it is possible that the adipose tissue phenotype of *Fos*Tg mice is a mere consequence of osteosarcoma progression [65]. Thus, we could show, by analyzing *Rsk2*-deficient *Fos* transgenic mice, that the reduced adipose tissue mass is linked to tumor development. Lack of Rsk2 in these mice led to a diminished tumor growth [32] causing a reduced body weight, adipose tissue mass and adipocyte phenotype intermediate to the phenotype of *Fos*Tg mice compared to wild-type mice. Furthermore, we could show that the level of Fgf21 is dependent on tumor size. In this context it is important to note, that Fgf21 is described to be secreted from several organs under stress conditions [66], and it is especially noteworthy that - while we observed an increased expression level of *Fgf21* in the liver, the main physiological site of Fgf21 production - we found that *Fgf21* expression is even further upregulated in osteosarcoma tissue. Therefore, while we proof an increase in Fgf21 production in *Fos*Tg mice, we do not demonstrate which site of expression is responsible for the observed metabolic phenotype.

Overall, cachexia is associated with a poor prognosis and increased mortality [67]. It has far-reaching negative effects on the quality of life and the treatment of cancer patients by impairing the tolerability and efficacy of cancer therapies [68–70]. Cachexia represents a major challenge in oncology treatment. A multimodal approach is recommended, which includes nutrition, physical activity and pharmacological interventions [71]. The detected elevated Fgf21 levels in patients with cardiac cachexia and cachexic geriatric patients [39, 72, 73] supports the use of Fgf21 as a biomarker for tumors with poor prognosis. On the other hand, as Fgf21 has been described to exert protective functions in cancer and cachexia [74], the elevated levels of Fgf21 might in our model reflect a compensatory response in *Fos*Tg mice to limit the loss of adipose tissue mass caused by tumor-induced cachexia and to ensure stable energy supply. This counteraction could be supported by the following reasons: (a) Fgf21 expression is induced during starvation affecting various tissues to overcome the deprivation status and (b) inhibition of Fgf21 was described to lead to a reduced muscle loss in cachexia [42]. Thus, better understanding the interaction of factors, such as Fos, Fgf21 and Ppars in the regulation of insulin, lipid metabolism and cancer associated cachexia can contribute to the identification of novel therapeutic targets for the treatment of cancer-associated metabolic syndromes.

In conclusion, our study not only shows that Fos is an important factor for the regulation of adipose tissue mass, glucose tolerance and insulin sensitivity as well as hepatic lipogenesis, but also that ubiquitous overexpression of Fos causes tumor-dependent Fgf21 activation. Thus, we suggest that the induction of Fgf21 expression by Fos-induced tumor formation is the key event modulating the metabolic phenotype associated with cancer cachexia.

## Supplementary Information

Below is the link to the electronic supplementary material.

Supplementary Material 1

Supplementary Material 2

## Data Availability

The dataset that supports the findings of this study is available from the corresponding author upon request.

## References

[1] Eferl R, Wagner EF. AP-1: a double-edged sword in tumorigenesis. Nat Rev Cancer. 2003;3(11):859–68. 14668816 10.1038/nrc1209

[2] Zenz R, Eferl R, Scheinecker C, Redlich K, Smolen J, Schonthaler HB, et al. Activator protein 1 (Fos/Jun) functions in inflammatory bone and skin disease. Arthritis Res Ther. 2008;10(1):201. 18226189 10.1186/ar2338 PMC2374460

[3] Jochum W, David JP, Elliott C, Wutz A, Plenk H Jr., Matsuo K, et al. Increased bone formation and osteosclerosis in mice overexpressing the transcription factor Fra-1. Nat Med. 2000;6(9):980–4. 10973316 10.1038/79676

[4] Sabatakos G, Sims NA, Chen J, Aoki K, Kelz MB, Amling M, et al. Overexpression of DeltaFosB transcription factor(s) increases bone formation and inhibits adipogenesis. Nat Med. 2000;6(9):985–90. 10973317 10.1038/79683

[5] Bozec A, Bakiri L, Jimenez M, Schinke T, Amling M, Wagner EF. Fra-2/AP-1 controls bone formation by regulating osteoblast differentiation and collagen production. J Cell Biol. 2010;190(6):1093–106. 20837772 10.1083/jcb.201002111 PMC3101588

[6] Eferl R, Hoebertz A, Schilling AF, Rath M, Karreth F, Kenner L, et al. The Fos-related antigen Fra-1 is an activator of bone matrix formation. EMBO J. 2004;23(14):2789–99. 15229648 10.1038/sj.emboj.7600282 PMC514946

[7] Grigoriadis AE, Schellander K, Wang ZQ, Wagner EF. Osteoblasts are target cells for transformation in c-fos Transgenic mice. J Cell Biol. 1993;122(3):685–701. 8335693 10.1083/jcb.122.3.685 PMC2119671

[8] Curran T, Peters G, Van Beveren C, Teich NM, Verma IM. FBJ murine osteosarcoma virus: identification and molecular cloning of biologically active proviral DNA. J Virol. 1982;44(2):674–82. 6292525 10.1128/jvi.44.2.674-682.1982 PMC256311

[9] Gamberi G, Benassi MS, Bohling T, Ragazzini P, Molendini L, Sollazzo MR, et al. C-myc and c-fos in human osteosarcoma: prognostic value of mRNA and protein expression. Oncology. 1998;55(6):556–63. 9778623 10.1159/000011912

[10] Wu JX, Carpenter PM, Gresens C, Keh R, Niman H, Morris JW, et al. The proto-oncogene c-fos is over-expressed in the majority of human osteosarcomas. Oncogene. 1990;5(7):989–1000. 2115647

[11] Argiles JM, Busquets S, Stemmler B, Lopez-Soriano FJ. Cancer cachexia: Understanding the molecular basis. Nat Rev Cancer. 2014;14(11):754–62. 25291291 10.1038/nrc3829

[12] Konsman JP, Blomqvist A. Forebrain patterns of c-Fos and FosB induction during cancer-associated anorexia-cachexia in rat. Eur J Neurosci. 2005;21(10):2752–66. 15926923 10.1111/j.1460-9568.2005.04102.x

[13] Sun Q, van de Lisdonk D, Ferrer M, Gegenhuber B, Wu M, Park Y, et al. Area Postrema neurons mediate interleukin-6 function in cancer cachexia. Nat Commun. 2024;15(1):4682. 38824130 10.1038/s41467-024-48971-1 PMC11144211

[14] Judge SM, Wu CL, Beharry AW, Roberts BM, Ferreira LF, Kandarian SC, et al. Genome-wide identification of FoxO-dependent gene networks in skeletal muscle during C26 cancer cachexia. BMC Cancer. 2014;14:997. 25539728 10.1186/1471-2407-14-997 PMC4391468

[15] Moore-Carrasco R, Busquets S, Almendro V, Palanki M, Lopez-Soriano FJ, Argiles JM. The AP-1/NF-kappaB double inhibitor SP100030 can revert muscle wasting during experimental cancer cachexia. Int J Oncol. 2007;30(5):1239–45. 17390027

[16] Barutcu AR, Elizalde G, Gonzalez AE, Soni K, Rinn JL, Wagers AJ, et al. Prolonged FOS activity disrupts a global myogenic transcriptional program by altering 3D chromatin architecture in primary muscle progenitor cells. Skelet Muscle. 2022;12(1):20. 35971133 10.1186/s13395-022-00303-x PMC9377060

[17] Moore-Carrasco R, Garcia-Martinez C, Busquets S, Ametller E, Barreiro E, Lopez-Soriano FJ, et al. The AP-1/CJUN signaling cascade is involved in muscle differentiation: implications in muscle wasting during cancer cachexia. FEBS Lett. 2006;580(2):691–6. 16412434 10.1016/j.febslet.2005.12.084

[18] Zhu S, Yang N, Guan Y, Wang X, Zang G, Lv X, et al. GDF15 promotes glioma stem cell-like phenotype via regulation of ERK1/2-c-Fos-LIF signaling. Cell Death Discov. 2021;7(1):3. 33431816 10.1038/s41420-020-00395-8 PMC7801449

[19] Jagodzinska A, Chudecka-Glaz A, Michalczyk K, Pius-Sadowska E, Wieder-Huszla S, Jurczak A, et al. The diagnostic role of FGF 21 in endometrial cancer and other pathologies of the uterine corpus. Diagnostics (Basel). 2023;13(3). 10.3390/diagnostics13030399 PMC9914808 36766504

[20] Kang YE, Kim JT, Lim MA, Oh C, Liu L, Jung SN, et al. Association between circulating fibroblast growth factor 21 and aggressiveness in thyroid cancer. Cancers (Basel). 2019;11(8). 10.3390/cancers11081154 PMC6721537 31408968

[21] Knott ME, Minatta JN, Roulet L, Gueglio G, Pasik L, Ranuncolo SM, et al. Circulating fibroblast growth factor 21 (Fgf21) as diagnostic and prognostic biomarker in renal cancer. J Mol Biomark Diagn. 2016;1(2). 10.4172/2155-9929.S2-015 PMC4922529 27358750

[22] Dai H, Hu W, Zhang L, Jiang F, Mao X, Yang G, et al. FGF21 facilitates autophagy in prostate cancer cells by inhibiting the PI3K-Akt-mTOR signaling pathway. Cell Death Dis. 2021;12(4):303. 33753729 10.1038/s41419-021-03588-w PMC7985321

[23] Luo Y, Yang Y, Liu M, Wang D, Wang F, Bi Y, et al. Oncogenic KRAS reduces expression of FGF21 in acinar cells to promote pancreatic tumorigenesis in mice on a High-Fat diet. Gastroenterology. 2019;157(5):1413–e2811. 31352001 10.1053/j.gastro.2019.07.030 PMC6815712

[24] Huang X, Yu C, Jin C, Yang C, Xie R, Cao D, et al. Forced expression of hepatocyte-specific fibroblast growth factor 21 delays initiation of chemically induced hepatocarcinogenesis. Mol Carcinog. 2006;45(12):934–42. 16929488 10.1002/mc.20241

[25] Yu X, Li Y, Jiang G, Fang J, You Z, Shao G, et al. FGF21 promotes non-small cell lung cancer progression by SIRT1/PI3K/AKT signaling. Life Sci. 2021;269:118875. 33310036 10.1016/j.lfs.2020.118875

[26] Hu C, Qiao W, Li X, Ning ZK, Liu J, Dalangood S, et al. Tumor-secreted FGF21 acts as an immune suppressor by rewiring cholesterol metabolism of CD8(+)T cells. Cell Metab. 2024;36(5):1168. 38537635 10.1016/j.cmet.2024.03.013

[27] Camporez JP, Jornayvaz FR, Petersen MC, Pesta D, Guigni BA, Serr J, et al. Cellular mechanisms by which FGF21 improves insulin sensitivity in male mice. Endocrinology. 2013;154(9):3099–109. 23766126 10.1210/en.2013-1191 PMC3749479

[28] Badman MK, Koester A, Flier JS, Kharitonenkov A, Maratos-Flier E. Fibroblast growth factor 21-deficient mice demonstrate impaired adaptation to ketosis. Endocrinology. 2009;150(11):4931–40. 19819944 10.1210/en.2009-0532 PMC2775979

[29] Kharitonenkov A, Shiyanova TL, Koester A, Ford AM, Micanovic R, Galbreath EJ, et al. FGF-21 as a novel metabolic regulator. J Clin Invest. 2005;115(6):1627–35. 15902306 10.1172/JCI23606 PMC1088017

[30] Ruther U, Komitowski D, Schubert FR, Wagner EF. c-fos expression induces bone tumors in Transgenic mice. Oncogene. 1989;4(7):861–5. 2547184

[31] Yang X, Matsuda K, Bialek P, Jacquot S, Masuoka HC, Schinke T, et al. ATF4 is a substrate of RSK2 and an essential regulator of osteoblast biology; implication for Coffin-Lowry syndrome. Cell. 2004;117(3):387–98. 15109498 10.1016/s0092-8674(04)00344-7

[32] David JP, Mehic D, Bakiri L, Schilling AF, Mandic V, Priemel M, et al. Essential role of RSK2 in c-Fos-dependent osteosarcoma development. J Clin Invest. 2005;115(3):664–72. 15719069 10.1172/JCI22877 PMC548699

[33] David JP, Neff L, Chen Y, Rincon M, Horne WC, Baron R. A new method to isolate large numbers of rabbit osteoclasts and osteoclast-like cells: application to the characterization of serum response element binding proteins during osteoclast differentiation. J Bone Miner Res. 1998;13:1730–8. 10.1359/jbmr.1998.13.11.1730 9797482

[34] David JP, Sabapathy K, Hoffmann O, Idarraga MH, Wagner EF. JNK1 modulates osteoclastogenesis through both c-Jun phosphorylation-dependent and -independent mechanisms. J Cell Sci. 2002;115:4317–25. 10.1242/jcs.00082 12376563

[35] Schwabe K, Garcia M, Ubieta K, Hannemann N, Herbort B, Luther J, et al. Inhibition of osteoarthritis by Adipose-Derived stromal cells overexpressing Fra-1 in mice. Arthritis Rheumatol. 2016;68(1):138–51. 26361381 10.1002/art.39425

[36] Luther J, Driessler F, Megges M, Hess A, Herbort B, Mandic V, et al. Elevated Fra-1 expression causes severe lipodystrophy. J Cell Sci. 2011;124(Pt 9):1465–76. 21486951 10.1242/jcs.079855

[37] Matsuo K, Owens JM, Tonko M, Elliott C, Chambers TJ, Wagner EF. Fosl1 is a transcriptional target of c-Fos during osteoclast differentiation. Nat Genet. 2000;24(2):184–7. 10655067 10.1038/72855

[38] Dai R, Wang Z, Samanipour R, Koo KI, Kim K. Adipose-Derived stem cells for tissue engineering and regenerative medicine applications. Stem Cells Int. 2016;2016:6737345. 27057174 10.1155/2016/6737345 PMC4761677

[39] Franz K, Ost M, Otten L, Herpich C, Coleman V, Endres AS, et al. Higher serum levels of fibroblast growth factor 21 in old patients with cachexia. Nutrition. 2019;63–4:81–6. 10.1016/j.nut.2018.11.004 30933730

[40] Fearon K, Strasser F, Anker SD, Bosaeus I, Bruera E, Fainsinger RL, et al. Definition and classification of cancer cachexia: an international consensus. Lancet Oncol. 2011;12(5):489–95. 21296615 10.1016/S1470-2045(10)70218-7

[41] Larson KR, Jayakrishnan D, Soto Sauza KA, Goodson ML, Chaffin AT, Davidyan A, et al. FGF21 induces skeletal muscle atrophy and increases amino acids in female mice: A potential role for glucocorticoids. Endocrinology. 2024;165(3). 10.1210/endocr/bqae004 PMC10849119 38244215

[42] Oost LJ, Kustermann M, Armani A, Blaauw B, Romanello V. Fibroblast growth factor 21 controls mitophagy and muscle mass. J Cachexia Sarcopenia Muscle. 2019;10(3):630–42. 30895728 10.1002/jcsm.12409 PMC6596457

[43] Potthoff MJ, Inagaki T, Satapati S, Ding X, He T, Goetz R, et al. FGF21 induces PGC-1alpha and regulates carbohydrate and fatty acid metabolism during the adaptive starvation response. Proc Natl Acad Sci U S A. 2009;106(26):10853–8. 19541642 10.1073/pnas.0904187106 PMC2705613

[44] Eskiler GG, Bezdegumeli E, Ozman Z, Ozkan AD, Bilir C, Kucukakca BN, et al. IL-6 mediated JAK/STAT3 signaling pathway in cancer patients with cachexia. Bratisl Lek Listy. 2019;66(11):819–26. 31747761 10.4149/BLL_2019_136

[45] Peng T, Zhang T, Lu X, Feng Q. JNK1/c-fos inhibits cardiomyocyte TNF-alpha expression via a negative crosstalk with ERK and p38 MAPK in endotoxaemia. Cardiovasc Res. 2009;81(4):733–41. 19043087 10.1093/cvr/cvn336

[46] Pinent M, Prokesch A, Hackl H, Voshol PJ, Klatzer A, Walenta E, et al. Adipose triglyceride lipase and hormone-sensitive lipase are involved in fat loss in JunB-deficient mice. Endocrinology. 2011;152(7):2678–89. 21540289 10.1210/en.2010-1477 PMC3152802

[47] Luther J, Ubieta K, Hannemann N, Jimenez M, Garcia M, Zech C, et al. Fra-2/AP-1 controls adipocyte differentiation and survival by regulating PPARgamma and hypoxia. Cell Death Differ. 2014;21(4):655–64. 24464219 10.1038/cdd.2013.198 PMC3950327

[48] Kveiborg M, Sabatakos G, Chiusaroli R, Wu M, Philbrick WM, Horne WC, et al. DeltaFosB induces osteosclerosis and decreases adipogenesis by two independent cell-autonomous mechanisms. Mol Cell Biol. 2004;24(7):2820–30. 15024071 10.1128/MCB.24.7.2820-2830.2004 PMC371096

[49] Rowe GC, Choi CS, Neff L, Horne WC, Shulman GI, Baron R. Increased energy expenditure and insulin sensitivity in the high bone mass DeltaFosB Transgenic mice. Endocrinology. 2009;150(1):135–43. 18772235 10.1210/en.2008-0678 PMC2630902

[50] Ruther U. Induction of bone tumors in Transgenic mice by C-FOS depends on the presence of a retroviral long terminal repeat. Cancer Genet Cytogenet. 1998;105(2):123–7. 9723028 10.1016/s0165-4608(98)00024-7

[51] Hasenfuss SC, Bakiri L, Thomsen MK, Williams EG, Auwerx J, Wagner EF. Regulation of steatohepatitis and PPARgamma signaling by distinct AP-1 dimers. Cell Metab. 2014;19(1):84–95. 24411941 10.1016/j.cmet.2013.11.018 PMC4023468

[52] Muise ES, Azzolina B, Kuo DW, El-Sherbeini M, Tan Y, Yuan X, et al. Adipose fibroblast growth factor 21 is up-regulated by peroxisome proliferator-activated receptor gamma and altered metabolic States. Mol Pharmacol. 2008;74(2):403–12. 18467542 10.1124/mol.108.044826

[53] Inagaki T, Dutchak P, Zhao G, Ding X, Gautron L, Parameswara V, et al. Endocrine regulation of the fasting response by PPARalpha-mediated induction of fibroblast growth factor 21. Cell Metab. 2007;5(6):415–25. 17550777 10.1016/j.cmet.2007.05.003

[54] Xiao F, Guo Y, Deng J, Yuan F, Xiao Y, Hui L, et al. Hepatic c-Jun regulates glucose metabolism via FGF21 and modulates body temperature through the neural signals. Mol Metab. 2019;20:138–48. 30579932 10.1016/j.molmet.2018.12.003 PMC6358569

[55] Zhang Y, Le Y, Ji Y, Yarde S, Yu X, Cheng X. Activation of activator protein-1-fibroblast growth factor 21 signaling attenuates cisplatin hepatotoxicity. Biochem Pharmacol. 2021;194:114823. 34748822 10.1016/j.bcp.2021.114823

[56] Huang Z, Zhong L, Zhu J, Xu H, Ma W, Zhang L, et al. Inhibition of IL-6/JAK/STAT3 pathway rescues denervation-induced skeletal muscle atrophy. Ann Transl Med. 2020;8(24):1681. 33490193 10.21037/atm-20-7269 PMC7812230

[57] Bonetto A, Aydogdu T, Jin X, Zhang Z, Zhan R, Puzis L, et al. JAK/STAT3 pathway Inhibition blocks skeletal muscle wasting downstream of IL-6 and in experimental cancer cachexia. Am J Physiol Endocrinol Metab. 2012;303(3):E410–21. 22669242 10.1152/ajpendo.00039.2012 PMC3423125

[58] Arora G, Gupta A, Guo T, Gandhi A, Laine A, Williams D, et al. JAK inhibitors suppress cancer Cachexia-Associated anorexia and adipose wasting in mice. JCSM Rapid Commun. 2020;3(2):115–28. 33103159 10.1002/rco2.24 PMC7580845

[59] Vernia S, Cavanagh-Kyros J, Garcia-Haro L, Sabio G, Barrett T, Jung DY, et al. The PPARalpha-FGF21 hormone axis contributes to metabolic regulation by the hepatic JNK signaling pathway. Cell Metab. 2014;20(3):512–25. 25043817 10.1016/j.cmet.2014.06.010 PMC4156535

[60] Kang SG, Lee SE, Choi MJ, Chang JY, Kim JT, Zhang BY, et al. Th2 cytokines increase the expression of fibroblast growth factor 21 in the liver. Cells. 2021;10(6). 10.3390/cells10061298 PMC8225035 34073755

[61] Zhang X, Ding X, Wang C, Le Q, Wu D, Song A, et al. Depletion of JunB increases adipocyte thermogenic capacity and ameliorates diet-induced insulin resistance. Nat Metab. 2024;6(1):78–93. 38191667 10.1038/s42255-023-00945-1 PMC10954369

[62] Cai M, Ye H, Zhu X, Li X, Cai L, Jin J, et al. Fibroblast growth factor 21 relieves Lipopolysaccharide-Induced acute lung injury by suppressing JAK2/STAT3 signaling pathway. Inflammation. 2024;47(1):209–26. 37864659 10.1007/s10753-023-01905-3 PMC10799097

[63] Rauscher FJ 3rd, Voulalas PJ, Franza BR Jr., Curran T. Fos and Jun bind cooperatively to the AP-1 site: reconstitution in vitro. Genes Dev. 1988;2(12B):1687–99. 2467839 10.1101/gad.2.12b.1687

[64] Bakiri L, Matsuo K, Wisniewska M, Wagner EF, Yaniv M. Promoter specificity and biological activity of tethered AP-1 dimers. Mol Cell Biol. 2002;22(13):4952–64. 12052899 10.1128/MCB.22.13.4952-4964.2002 PMC133900

[65] Petruzzelli M, Wagner EF. Mechanisms of metabolic dysfunction in cancer-associated cachexia. Genes Dev. 2016;30(5):489–501. 26944676 10.1101/gad.276733.115 PMC4782044

[66] Luo Y, McKeehan WL. Stressed liver and muscle call on adipocytes with FGF21. Front Endocrinol (Lausanne). 2013;4:194. 24385972 10.3389/fendo.2013.00194 PMC3866528

[67] Arends J, Baracos V, Bertz H, Bozzetti F, Calder PC, Deutz NEP, et al. ESPEN expert group recommendations for action against cancer-related malnutrition. Clin Nutr. 2017;36(5):1187–96. 28689670 10.1016/j.clnu.2017.06.017

[68] da Rocha IMG, Marcadenti A, de Medeiros GOC, Bezerra RA, Rego JFM, Gonzalez MC, et al. Is cachexia associated with chemotherapy toxicities in Gastrointestinal cancer patients? A prospective study. J Cachexia Sarcopenia Muscle. 2019;10(2):445–54. 30924270 10.1002/jcsm.12391 PMC6463470

[69] Furuse J, Osugi F, Machii K, Niibe K, Endo T. Effect of cancer cachexia on first-line chemotherapy in patients with advanced pancreatic cancer: a claims database study in Japan. Int J Clin Oncol. 2024;29(4):456–63. 38353906 10.1007/s10147-024-02467-6 PMC10963515

[70] Prado CM, Baracos VE, McCargar LJ, Mourtzakis M, Mulder KE, Reiman T, et al. Body composition as an independent determinant of 5-fluorouracil-based chemotherapy toxicity. Clin Cancer Res. 2007;13(11):3264–8. 17545532 10.1158/1078-0432.CCR-06-3067

[71] Roeland EJ, Bohlke K, Baracos VE, Bruera E, Del Fabbro E, Dixon S, et al. Management of cancer cachexia: ASCO guideline. J Clin Oncol. 2020;38(21):2438–53. 32432946 10.1200/JCO.20.00611

[72] Refsgaard Holm M, Christensen H, Rasmussen J, Johansen ML, Schou M, Faber J, et al. Fibroblast growth factor 21 in patients with cardiac cachexia: a possible role of chronic inflammation. ESC Heart Fail. 2019;6(5):983–91. 31429530 10.1002/ehf2.12502 PMC6816069

[73] Conte M, Ostan R, Fabbri C, Santoro A, Guidarelli G, Vitale G, et al. Human aging and longevity are characterized by high levels of mitokines. J Gerontol Biol Sci Med Sci. 2019;74(5):600–7. 10.1093/gerona/gly153 29955888

[74] Lu W, Li X, Luo Y. FGF21 in obesity and cancer: new insights. Cancer Lett. 2021;499:5–13. 33264641 10.1016/j.canlet.2020.11.026 PMC7779663

