# Supplementary material for: Fos-induced osteosarcoma growth causes a cachexia-like phenotype in mice and correlates with high Fgf21 serum levels
Source: Cancer Metab. 2026 Feb 3;14:18. doi: 10.1186/s40170-025-00417-y (PMC13218021; doi:10.1186/s40170-025-00417-y)
Supplement: Supplementary file 1 — Supplementary Material 1 [file 40170_2025_417_MOESM1_ESM.pdf]

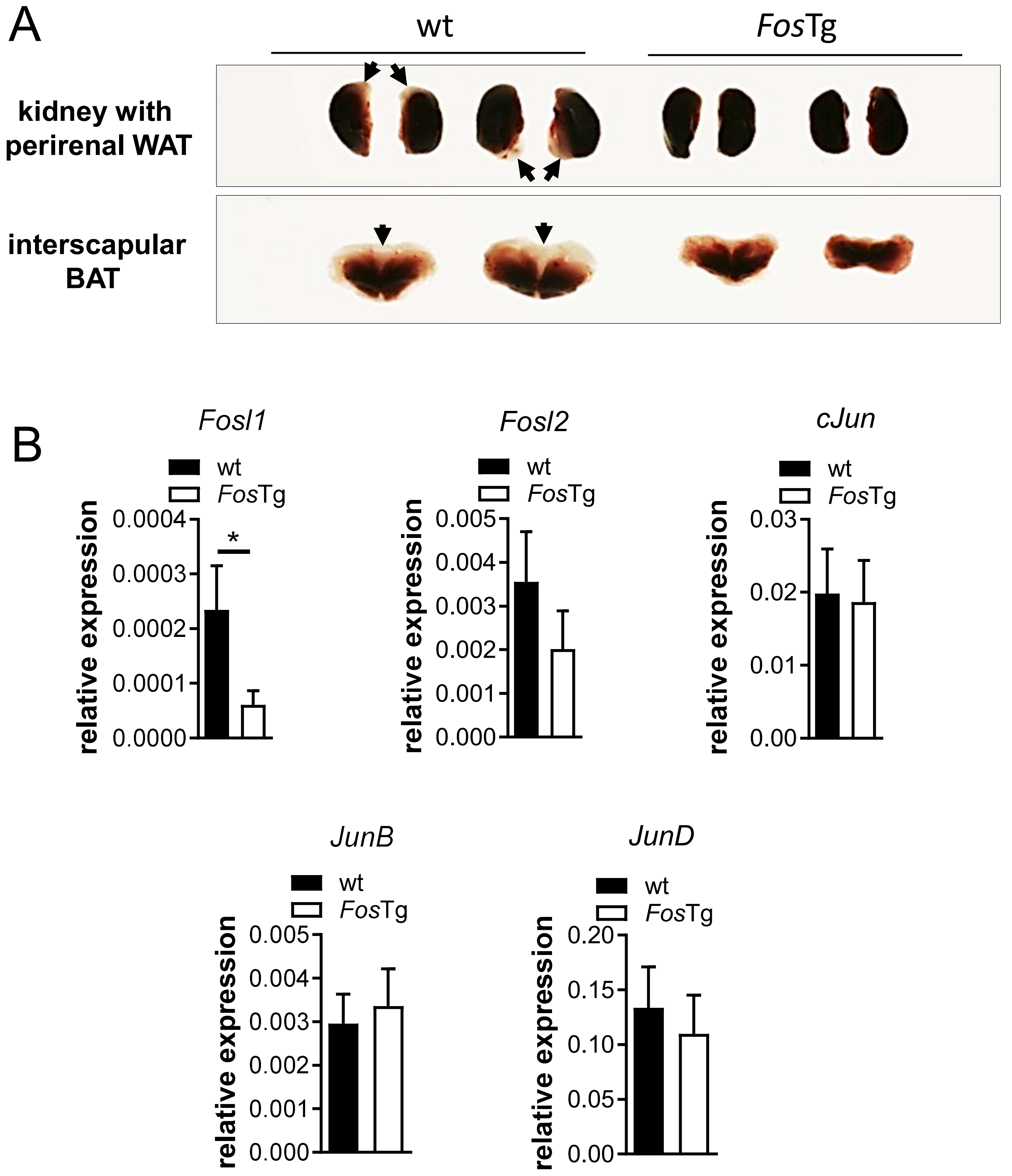

Figure S1. ***Fos-induced reduction of adipose tissue mass is not associated with an increased expression of other AP-1 members.*** (A) Representative images of the perirenal WAT in the wt mice (indicated by the arrows) and of the interscapular BAT (the arrows indicate the presence of WAT in the wt mice) of 16-week-old wt and *Fos*Tg littermates. (B) Q-PCR analysis for expression of genes encoding AP-1 family members in the epididymal WAT of 16-week-old wt and *Fos*Tg mice. Data represent mean ± s.e.m. (n≥6). Asterisks indicate statistically significant differences (*$P < 0.05$).

Luther et al. Fig. S1

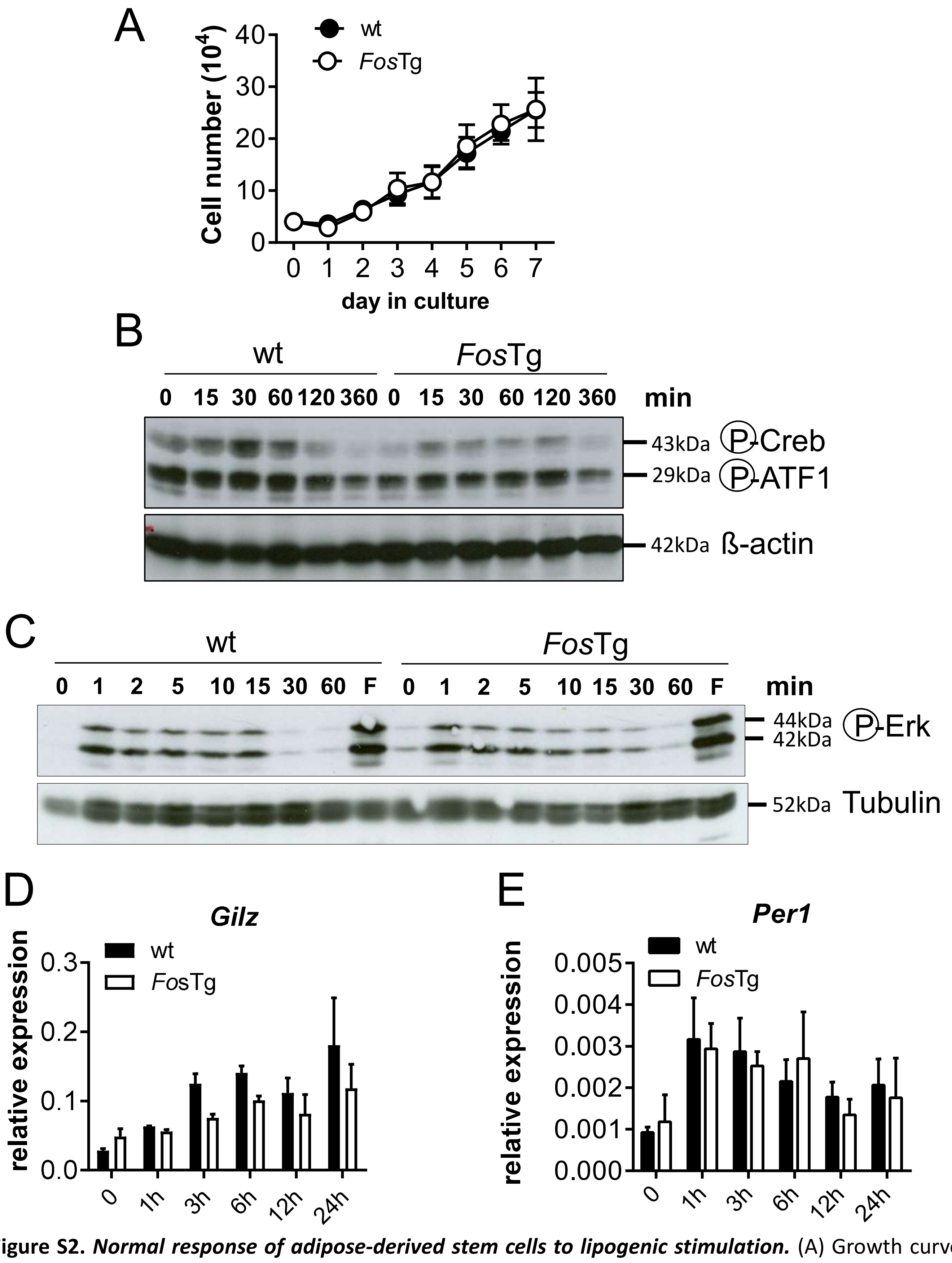

Figure S2. ***Normal response of adipose-derived stem cells to lipogenic stimulation.*** (A) Growth curve of ADSCs isolated from subcutaneous WAT of wild type (wt) and *Fos*Tg mice. The curve was established during the growing phase of the culture before induction of differentiation (n=3 independent cell isolations). (B) Western blot analysis of Creb and Atf1 phosphorylation in response to IBMX. (C) Western blot analysis of Erk phosphorylation in response to Insulin stimulation for 0 to 60 minutes; A 5-minute stimulation with FCS (indicated by F) was used as positive control. (D-E) Q-PCR analysis of *Gilz* (D) and *Per1* (E) induction in response to Dexamethasone stimulation for 0 to 24 hours. Data represent mean ± s.e.m. (n≥3).

Luther et al. Fig. S2

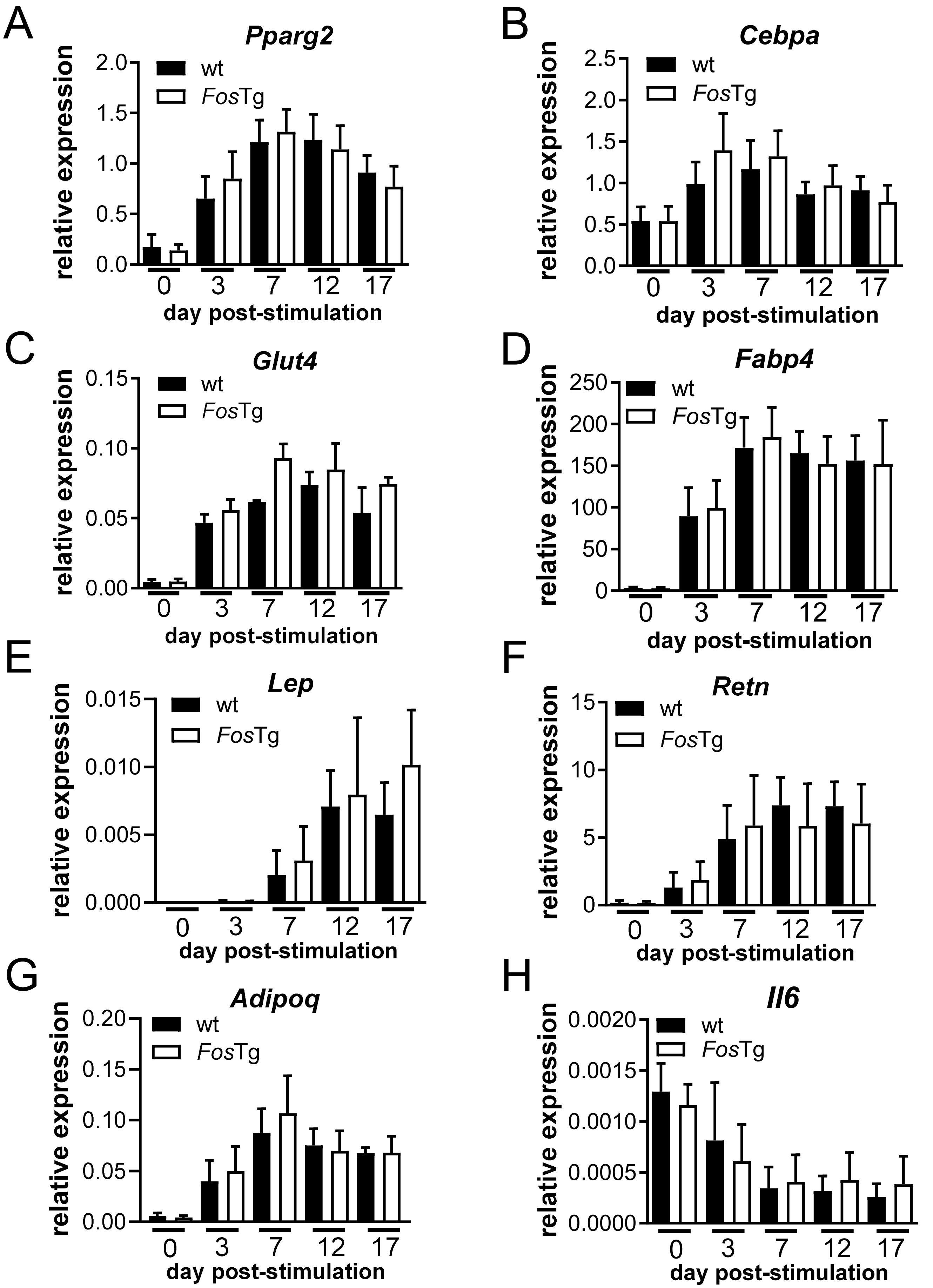

Figure S3. ***No change in markers for adipocyte differentiation and adipokine expression in cells isolated from Fos-transgenic mice compared to control.*** Q-PCR analyses for (A-D) adipogenic differentiation markers and (E-H) adipokines in wt and *Fos*Tg ADSCs during the course of adipocyte differentiation. Data represent mean + s.e.m. (n≥3 independent cell isolations).

Luther et al. Fig.S3

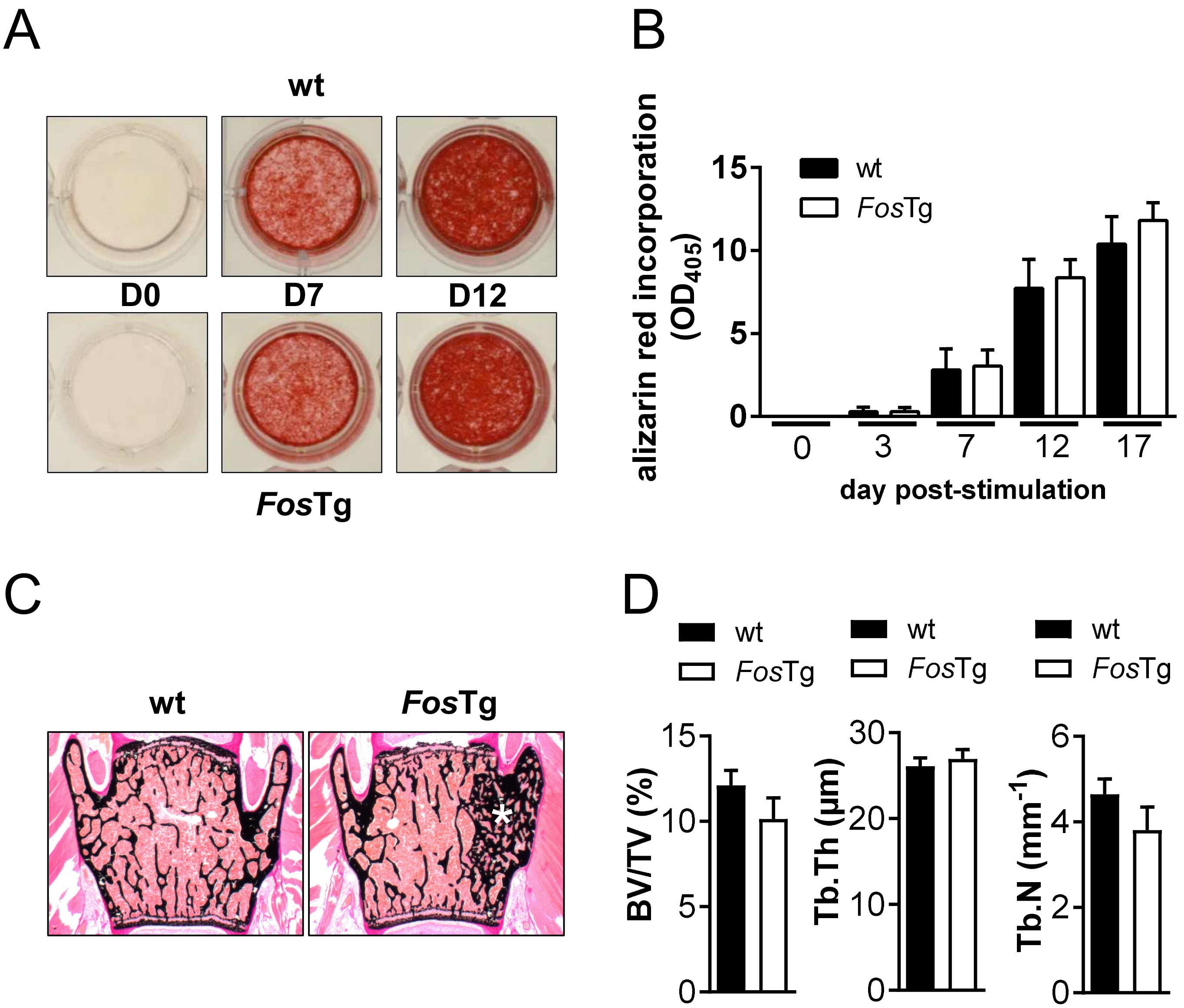

Figure S4. *No change in bone formation in Fos*Tg *mice.* (A) Representative images showing Alizarin Red staining of wt and *Fos*Tg ADSCs during the course of osteoblastic differentiation. (B) Quantification of alizarin red incorporation. (C) Von Kossa staining of vertebral body sections from 8-week-old wt and *Fos*Tg mice. The asterisk indicates the presence of a tumor. (D) Histomorphometric quantification of bone volume per tissue volume (BV/TV), trabecular thickness (Tb.Th.) and trabecular number (Tb.N.) in trabecular bone areas excluding the tumors. Data represent mean ± s.e.m. (n≥3).

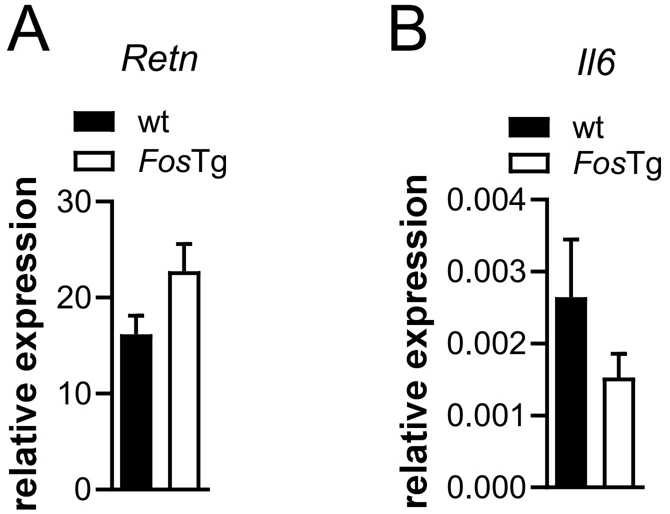

Figure S5. ***No significant change of adipokine expression in adipose tissue of Fos*****Tg** ***mice compared to wt.*** Q-PCR analyses of (A) resistin *(Retn)* and (B) *Il6* expression in gonadal adipose tissue of 16 week-old mice. Data represent mean $\pm$ s.e.m. (n≥3).

Luther et al. Fig. S5

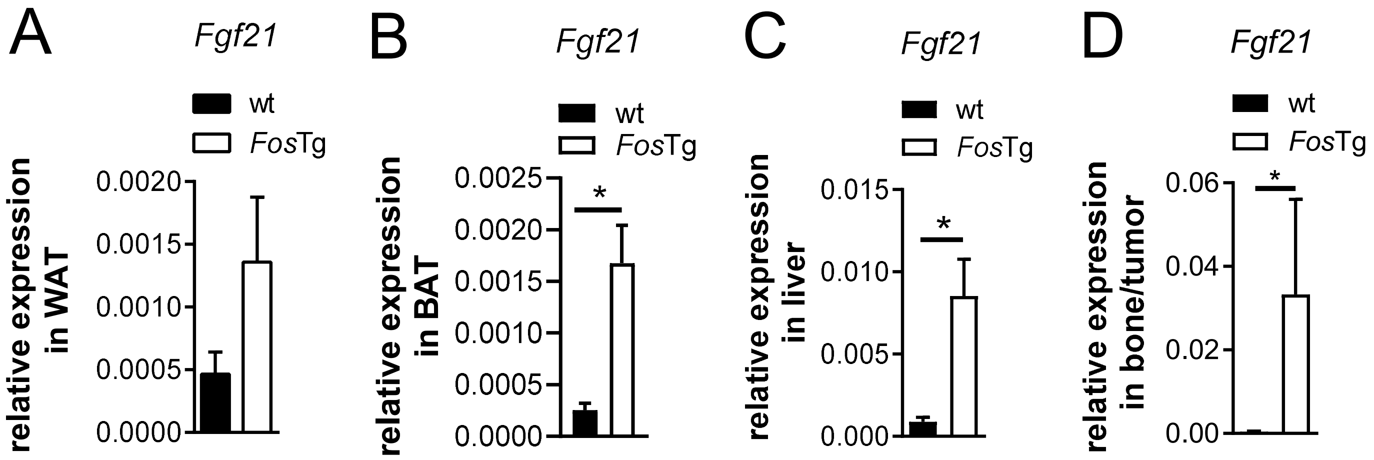

Figure S6. ***Significantly increased expression of Fgf21 in BAT, liver and tumor of FosTg mice compared to wt.*** Q-PCR analyses of *Fgf21* in (A) WAT, (B) BAT, (C) liver and (D) tumor tissue (compared to bone tissue of wt mice) of 16 week-old mice. While these results are also presented in Fig. 5F-G, the current representation shows the expression level in *Fos*Tg mice compared to the respective wt control. Data represent mean ± s.e.m. (n≥3). Asterisks indicate statistically significant differences (**P* < 0.05).

Luther et al. Fig. S6

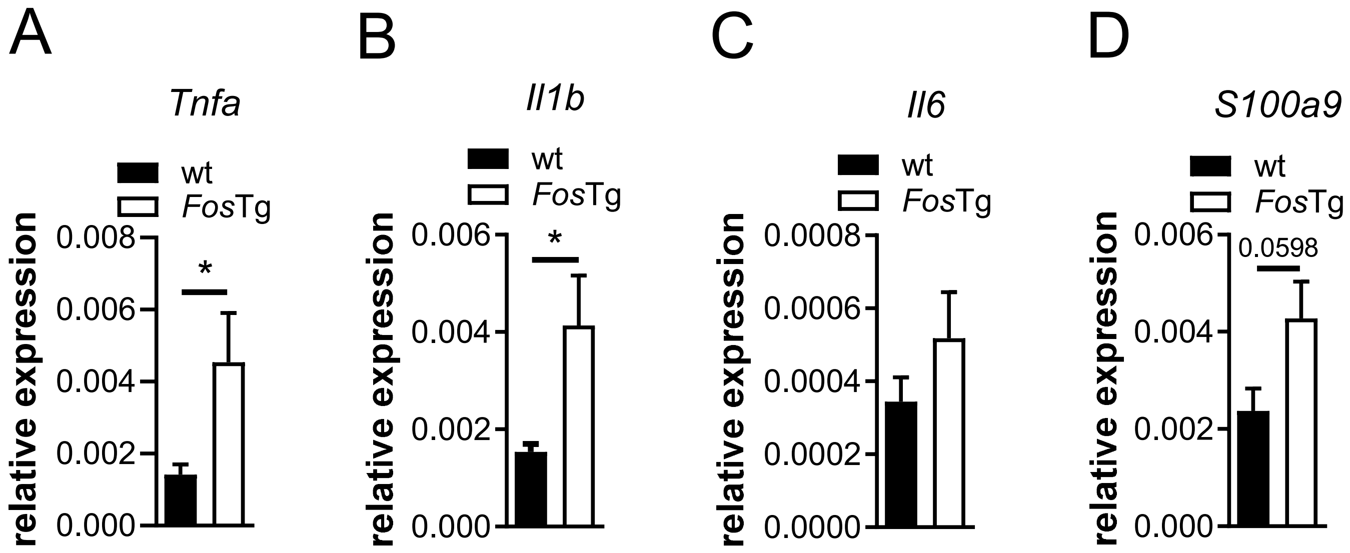

Figure S7. ***Increased expression of pro-inflammatory cytokines in the liver of FosTg mice compared to wt.*** Q-PCR analyses of (A) *Tnfa*, (B) *Il1b*, (C) *Il6* and (D) *S100a9* expression in the liver of 16 week-old mice. Data represent mean ± s.e.m. (n≥3). Asterisks indicate statistically significant differences (**P* < 0.05).

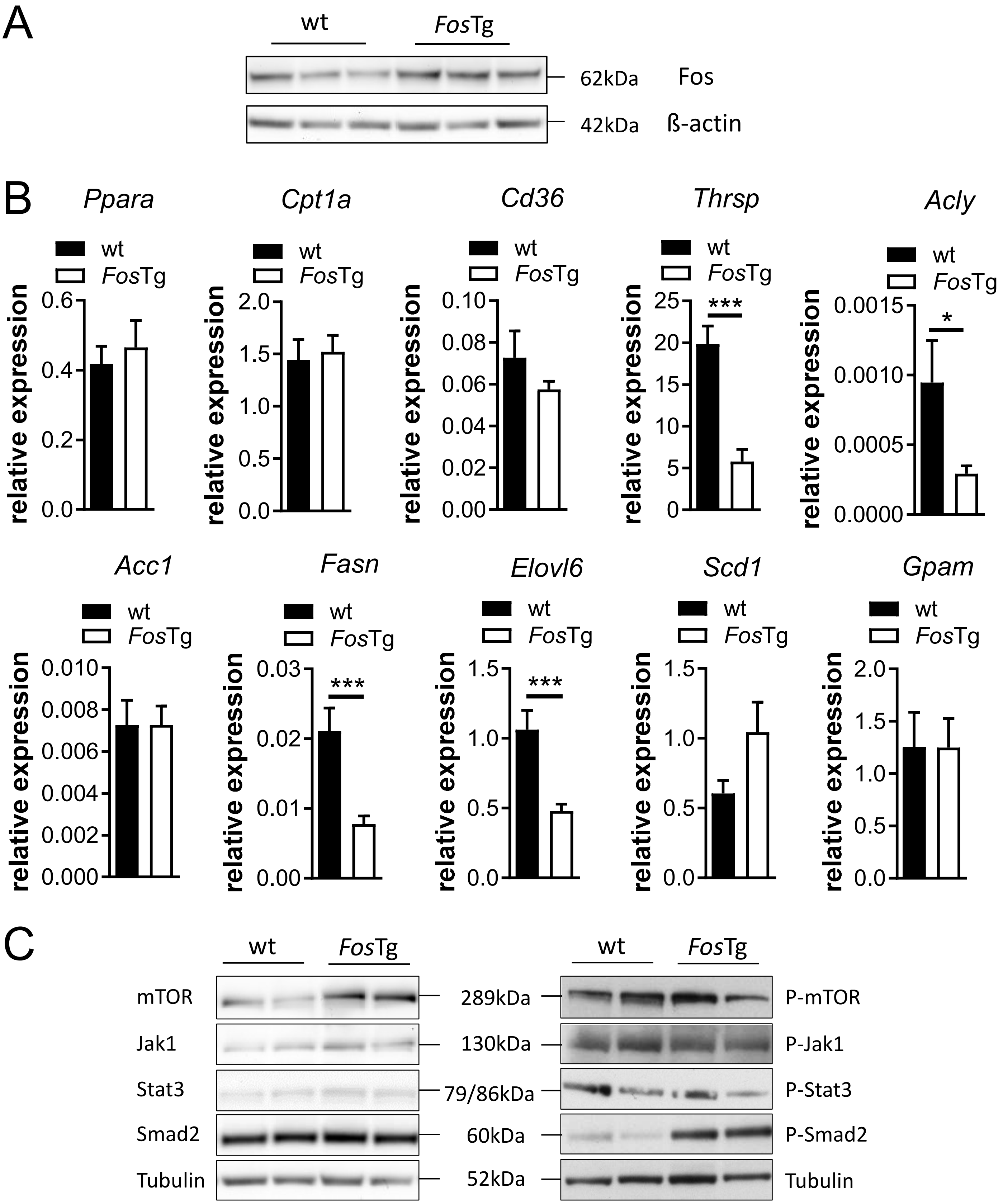

Figure S8*. Increased P-Smad2 levels in liver samples of FosTg mice.* (A) Western blot analysis of liver samples of 16-week-old *Fos*Tg compared to wt mice showing Fos protein levels (B) Q-PCR analyses of genes regulating lipogenesis. While these results are also presented in Fig. 7H, the current representation uses normalization solely to the housekeeping gene. (C) Western blot analysis of liver samples of 16-week-old *Fos*Tg compared to wt mice showing phosphorylated and non-phosphorylated levels of mTOR, Jak1, Stat3 and Smad2. Data represent mean $\pm$ s.e.m. (n≥3). Asterisks indicate statistically significant differences (**P* < 0.05; ****P* < 0.001).

Luther et al. Fig. S8

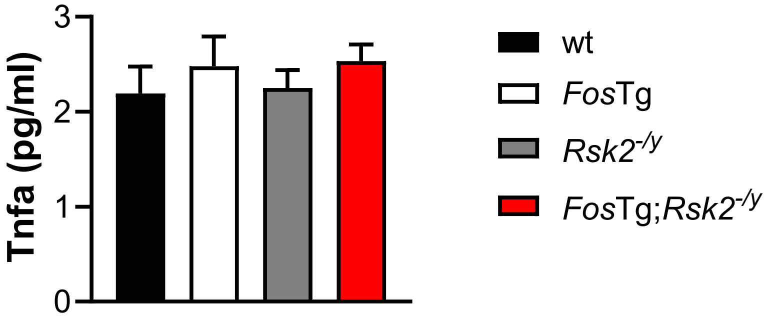

Figure S9. ***No change in serum Tnfa level in Fos*****Tg *mice.*** ELISA-based quantification of circulating Tnfa concentrations in 16-week-old mice of the indicated genotypes. Data represent mean ± s.e.m. (n≥3).

Luther et al. Fig. S9
