# Supplementary material for: Fos-induced osteosarcoma growth causes a cachexia-like phenotype in mice and correlates with high Fgf21 serum levels
Source: Cancer Metab. 2026 Feb 3;14:18. doi: 10.1186/s40170-025-00417-y (PMC13218021; doi:10.1186/s40170-025-00417-y)

Original western blot data of Fig. S2B

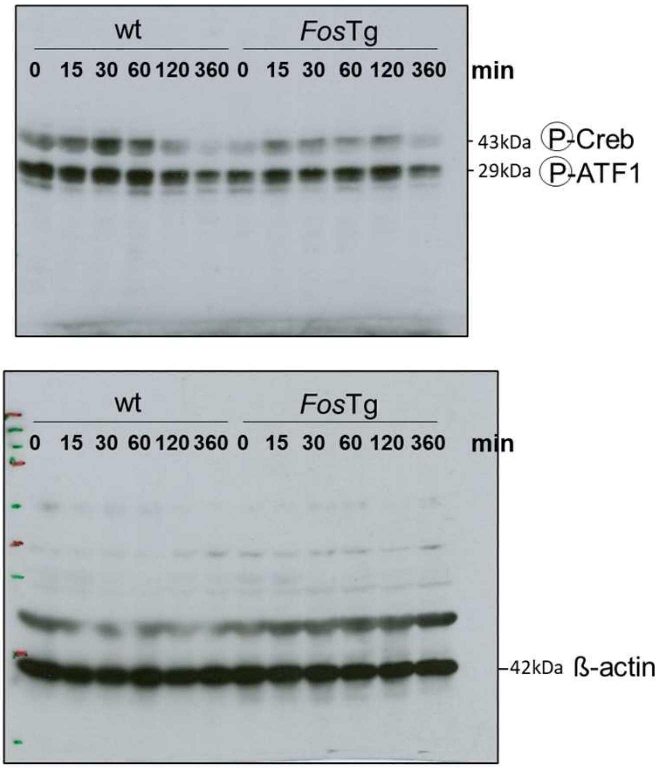

Original western blot data of Fig. S2C

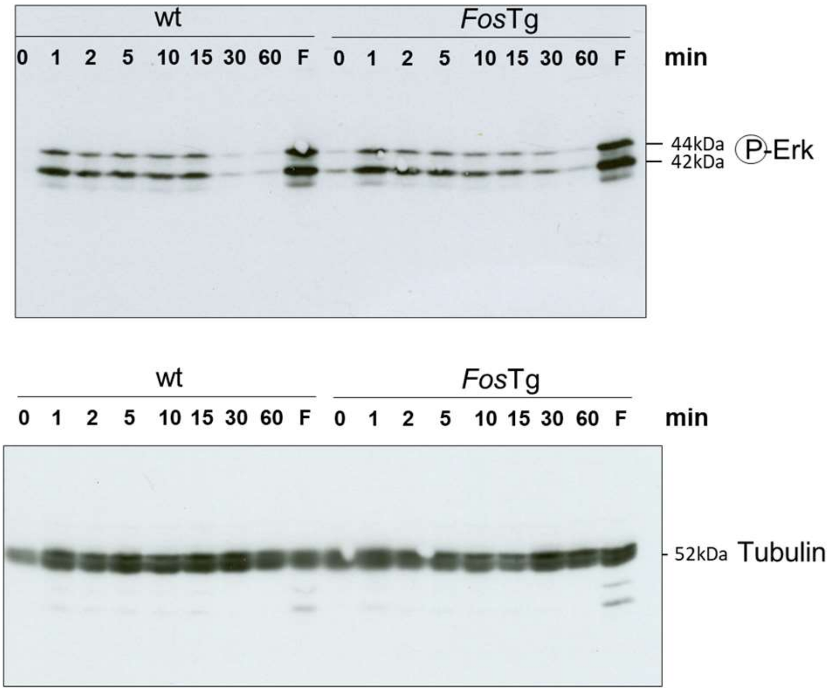

Original western blot data of Fig S8A

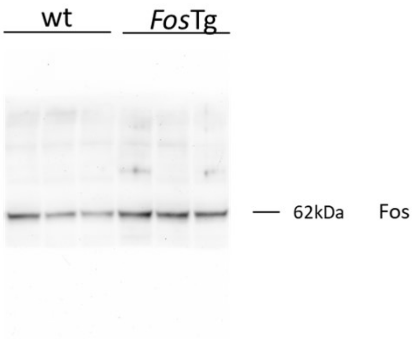

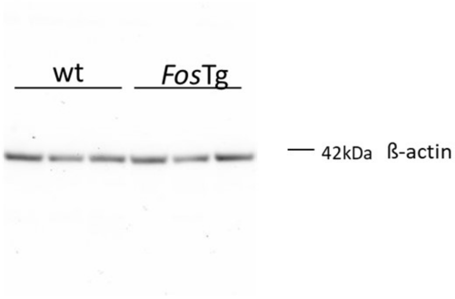

Original western blot data of Fig S8C

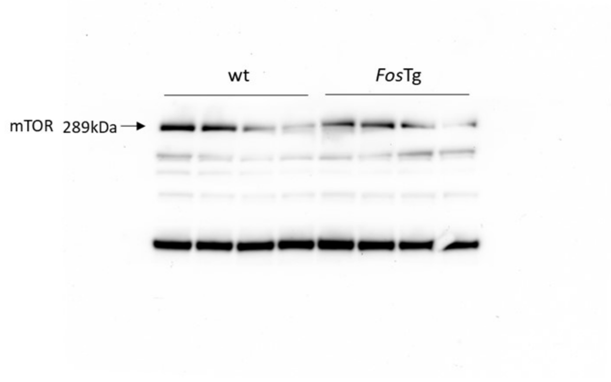

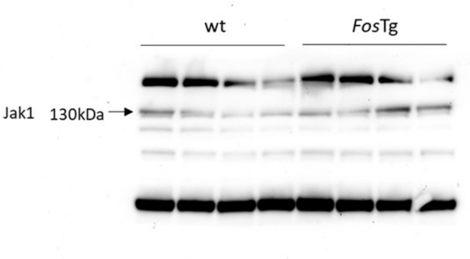

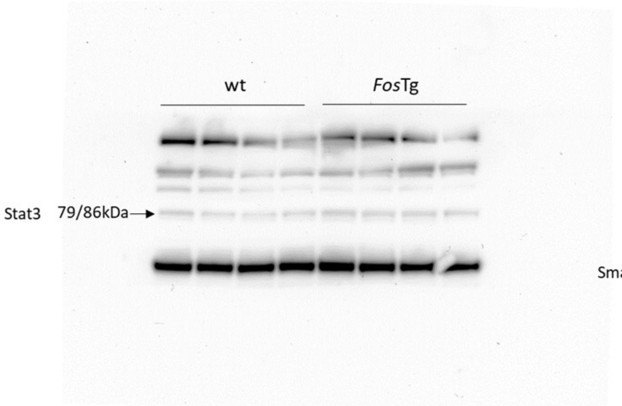

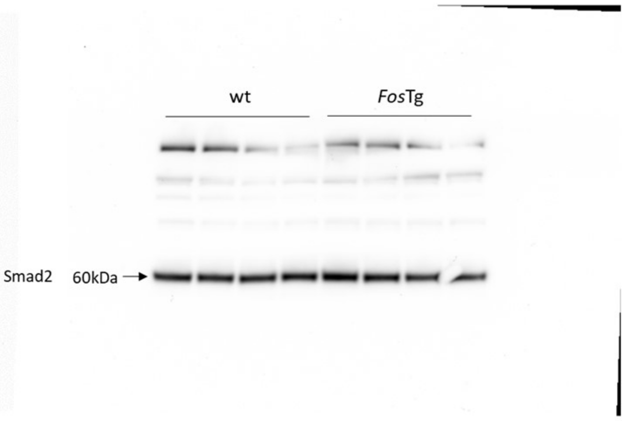

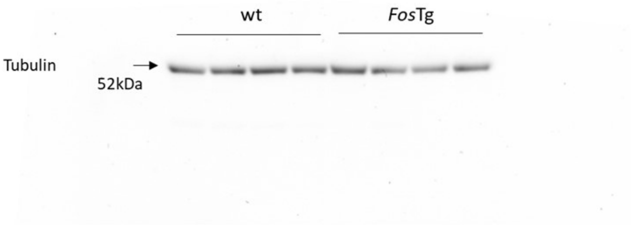

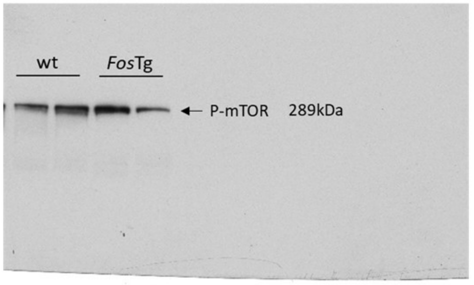

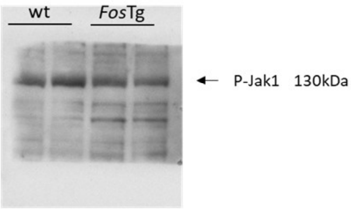

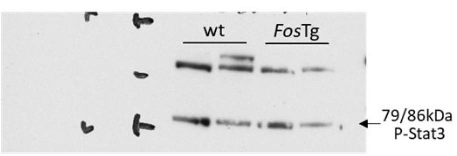

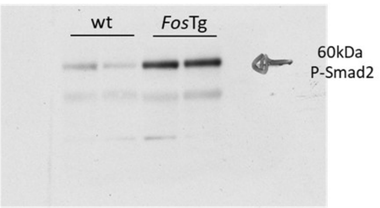

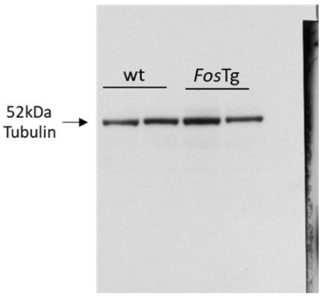

Supplement: Supplementary file 2 — Supplementary Material 2 [file 40170_2025_417_MOESM2_ESM.pdf]
